# CT-derived myocardial extracellular volume: current evidence, clinical utility, and the emerging role of photon-counting CT—a systematic review

**DOI:** 10.1186/s41747-026-00808-w

**Published:** 2026-09-22

**Authors:** Jacopo D’Argenzio, Francesco Stucchi, Valentina Tambè, Malaka Mammadova, Gianpaolo Carrafiello, Francesco Secchi

**Affiliations:** 1 Postgraduate School in Radiodiagnostics, Università Degli Studi Di Milano, Milan, Italy; 2 Cardiovascular Imaging Unit, IRCCS MultiMedica, Sesto San Giovanni, Milan, Italy; 3 Radiology Department, Fondazione IRCCS Cà Granda Ospedale Maggiore Policlinico, Milan, Italy; 4 Department of Oncology and Hematology-Oncology, Università Degli Studi Di Milano, Milan, Italy; 5 Department of Biomedical Sciences for Health, University of Milan, Milan, Italy

**Keywords:** Extracellular space, Fibrosis, Heart diseases, Myocardium, Tomography (x-ray computed)

## Abstract

**Objective:**

Cardiac extracellular volume (ECV) is a quantitative imaging biomarker of myocardial interstitial expansion and diffuse fibrosis, with cardiac magnetic resonance regarded as the reference standard method for assessment. Interest in computed tomography (CT)-derived cardiac ECV is increasing owing to its wide availability and potential integration into routine cardiac CT examinations. This systematic review was conducted within the framework of the European Network for the Assessment of Imaging in Medicine (EuroAIM).

**Materials and methods:**

This PRISMA-compliant systematic review searched PubMed, Embase, and Scopus, with the last search performed in December 2025. Extracted data included study design, target pathology, CT technology, acquisition approach, timing of delayed imaging, hematocrit estimation, region-of-interest methodology, type of comparator, and reporting and definition of ECV cutoff values. Study quality was evaluated using the Newcastle–Ottawa scale.

**Results:**

Of 304 unique records, 113 studies were included, with a marked increase in publications over the last 3 years (30 studies in 2025). Studies covered a broad range of clinical settings, including aortic stenosis, amyloidosis, heart failure, cardiomyopathies, myocarditis, and cardio-oncology. Sixty-seven studies were retrospective, and forty-six were prospective. ECV was assessed using single-energy CT (*n* = 65), dual-energy CT (*n* = 34), and photon-counting CT (*n* = 14, 10 of them in 2025). Cutoff values were reported in only 38 studies. The Newcastle–Ottawa Scale score was 6.33 ± 1.42 (mean ± standard deviation).

**Conclusion:**

Cardiac ECV is rapidly expanding, with photon-counting CT emerging as a promising platform. Nevertheless, substantial methodological heterogeneity and inconsistent cutoff reporting persist, highlighting the need for standardized protocols and harmonized reporting.

**Registration:**

PROSPERO CRD420251147762.

**Key Points:**

- ***Question*** What evidence is available in the literature about photon-counting CT-derived myocardial ECV quantification?
- ***Findings*** CT-derived myocardial ECV enables noninvasive assessment of diffuse myocardial fibrosis across multiple cardiovascular diseases.
- ***Relevance statement*** The rapidly expanding evidence and the emergence of photon-counting CT highlight its potential integration into routine cardiac CT imaging. Evidence spans multiple cardiac and systemic disease categories; heterogeneous methods limit cross-study comparability; standardized reporting is needed for clinical translation.

**Graphical Abstract:**

**Figure:**
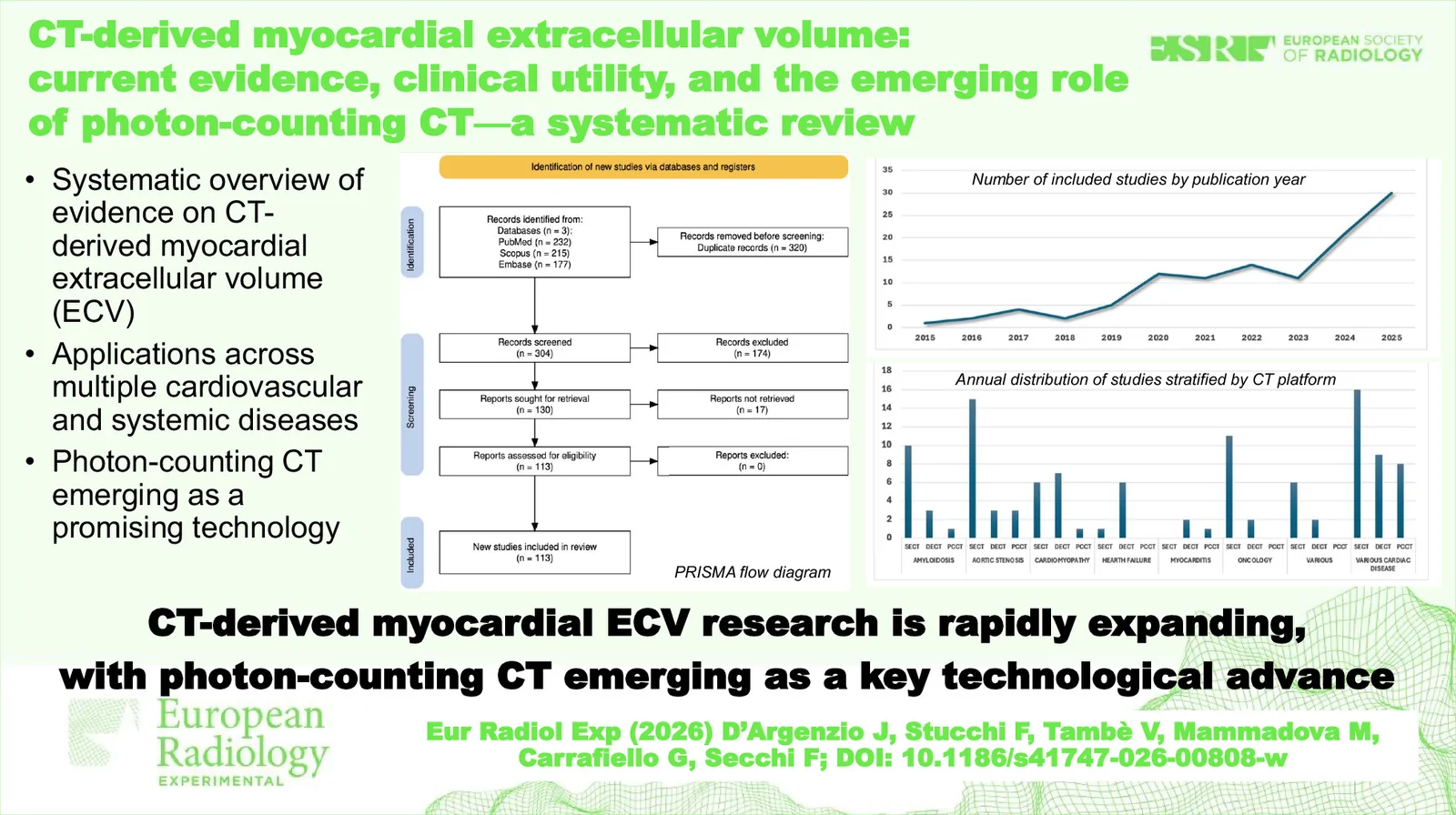

## Background

Myocardial interstitial expansion represents a common pathophysiological pathway across a broad spectrum of cardiovascular diseases, including cardiomyopathies, valvular heart disease, infiltrative disorders, inflammatory conditions, and systemic diseases with cardiac involvement. Diffuse myocardial fibrosis, a major component of interstitial expansion, contributes to adverse ventricular remodeling, impaired systolic and diastolic function, increased arrhythmic risk, and poorer clinical outcomes [1]. Consequently, the noninvasive characterization and quantification of myocardial interstitial changes have become increasingly relevant for diagnosis, risk stratification, and longitudinal disease monitoring.

Cardiac extracellular volume (ECV) has emerged as a quantitative imaging biomarker reflecting myocardial interstitial expansion and diffuse fibrosis. By estimating the fraction of myocardial tissue occupied by the extracellular space, ECV enables global myocardial tissue characterization beyond focal fibrosis detection. Cardiac magnetic resonance (CMR), using T1 mapping techniques before and after contrast administration, is currently regarded as the reference standard for ECV assessment and has been extensively validated across multiple cardiac pathologies [1]. CMR-derived myocardial extracellular volume (CMR-ECV) correlates with histological fibrosis, functional impairment, and adverse clinical outcomes. However, despite its diagnostic robustness, CMR presents practical limitations, including limited availability, longer examination times, higher costs, and contraindications related to implanted devices or patient instability, which may restrict its use in routine clinical workflows.

**Fig. 1:**
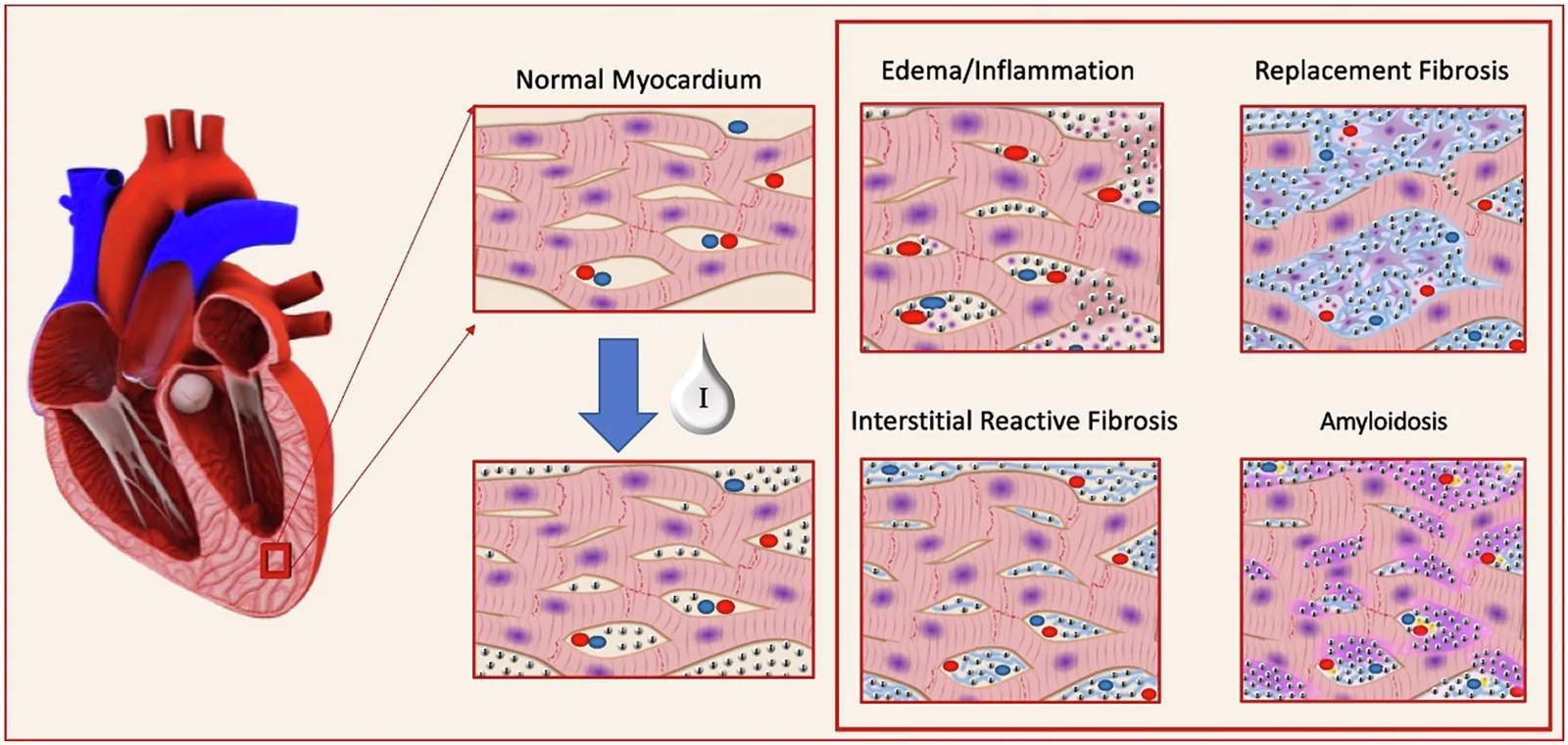
Conceptual schematic of myocardial tissue compartments and determinants of extracellular volume (ECV). The myocardium is composed of blood, intracellular, and extracellular compartments. Myocardial ECV reflects the fraction of tissue occupied by the extracellular space. Diffuse fibrosis, infiltrative processes (*e.g*., amyloidosis), interstitial edema, and myocyte loss lead to expansion of the extracellular compartment and result in increased ECV values

In parallel, computed tomography (CT) has evolved from a purely anatomical imaging modality into a platform capable of myocardial tissue characterization. The distribution properties of iodinated contrast agents within the extracellular space provide the physiological basis for CT-derived myocardial extracellular volume (CT-ECV) quantification (Fig. 1 [2]). With appropriate acquisition timing during the delayed equilibrium phase, myocardial iodine concentration can be used to estimate ECV through normalization to the blood pool and correction for hematocrit. It should be noted that ECV estimates may be influenced by contrast administration protocols and the timing of delayed image acquisition, as incomplete equilibrium between the intravascular and extracellular compartments can affect the accuracy and comparability of measurements [3]. Importantly, CT-based ECV assessment can be integrated into routine cardiac CT examinations, such as coronary CT angiography or pre-procedural planning for structural heart interventions.

**Fig. 2:**
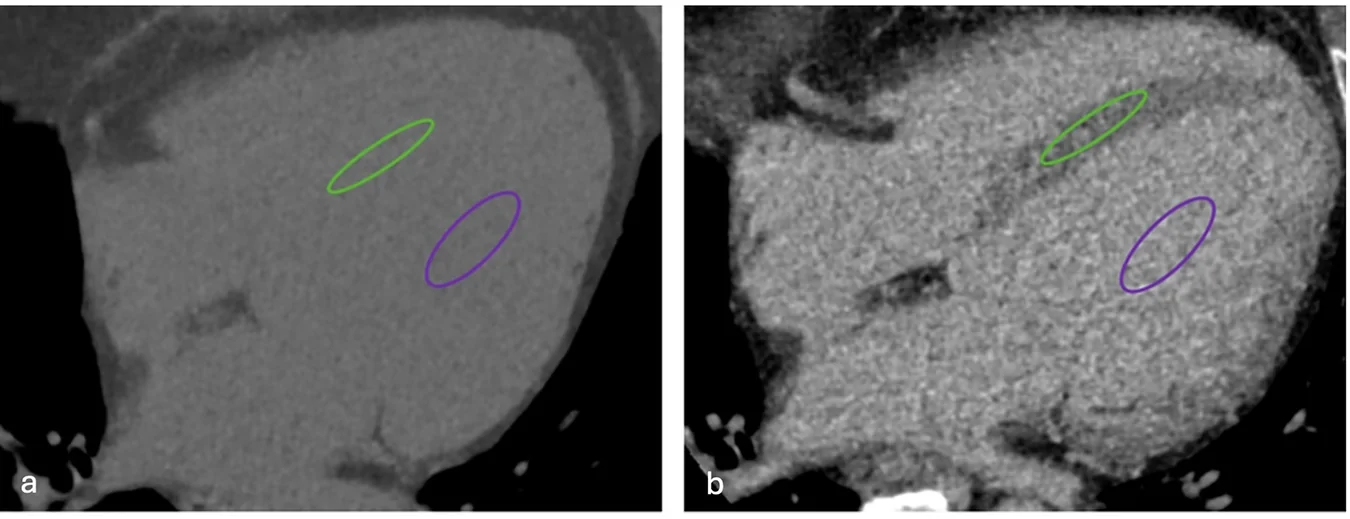
Example of myocardial extracellular volume (ECV) quantification on delayed-phase cardiac CT. Circular regions of interest (ROIs) are placed in the mid-ventricular septal myocardium and in the left ventricular blood pool on delayed contrast CT images. These ROIs are propagated to corresponding pre- (**a**) and post-contrast (**b**) scans to measure attenuation (Hounsfield units, HU)

Methodologically, CT-ECV is calculated using an approach analogous to that employed in CMR:

$${{{\rm{ECV}}}}=\left(1-{{{\rm{hematocrit}}}}\right)\times \left(\Delta {{{\rm{myocardium}}}}/\Delta {{{\rm{blood}}}}\right),$$

where Δ represents the change in myocardial and blood pool attenuation or iodine concentration between pre-contrast and delayed post-contrast acquisitions (Fig. 2 [4]). This method can be implemented using attenuation-based measurements in single-energy CT (SECT) or iodine density values in spectral CT techniques. However, variations in contrast administration, acquisition timing, region-of-interest definition, and reconstruction parameters may influence ECV estimates and contribute to inter-study variability. Several CT technologies have been investigated for ECV quantification.

Early studies relied on SECT, whereas dual-energy CT (DECT) enabled material decomposition and iodine mapping, improving the robustness of myocardial iodine quantification. Despite these advances, heterogeneity in acquisition and analysis strategies persists, limiting comparability across studies and the establishment of universally accepted ECV cutoff values.

More recently, photon-counting CT (PCCT) has emerged as a promising technology in cardiac imaging. Unlike conventional energy-integrating detectors, PCCT directly converts individual x-ray photons into electrical signals and intrinsically discriminates photon energies. This detector architecture enables higher spatial resolution, improved contrast-to-noise ratio, reduced electronic noise, and enhanced spectral fidelity, potentially improving iodine quantification and the reliability of ECV estimation. Beyond incremental improvements, PCCT may enable more accurate material separation and iodine quantification at lower contrast doses, potentially improving safety in patients with renal impairment. As PCCT technology matures and becomes more widely available, it may contribute to improved protocol harmonization and inter-vendor consistency in CT-ECV research [5].

Despite the rapidly expanding literature on CT-ECV, the available evidence remains heterogeneous. Studies differ in CT platforms, acquisition strategies, postprocessing techniques, and reporting of ECV cutoff values. Moreover, the contribution of emerging technologies such as PCCT has not yet been comprehensively synthesized. Therefore, a systematic review is warranted to summarize current evidence, highlight technological advances with particular emphasis on PCCT, and identify remaining challenges and future directions. This review was conducted under the umbrella of the European Network for the Assessment of Imaging in Medicine (EuroAIM; https://www.eibir.org/initiatives/euroaim/).

## Materials and methods

This systematic review was conducted according to the Preferred Reporting Items for Systematic Reviews and Meta-Analyses–PRISMA 2020 statement. The protocol was prospectively registered in the International Prospective Register of Systematic Reviews (PROSPERO; CRD420251147762).

A literature search was performed in PubMed, Embase, and Scopus for studies published between 1 January 2015 and 9 December 2025. No language restrictions were applied during the search; however, only studies available in English were included in the final analysis due to the feasibility of full-text assessment. Gray literature was not systematically explored, as the review focused on peer-reviewed studies indexed in major bibliographic databases. Search strategies used Boolean operators and database-specific syntax, combining terms related to extracellular volume, the heart, and computed tomography, including ECV, cardiac or myocardial tissue, CT, SECT, DECT, and PCCT. Complete search strategies are provided in the Supplementary Materials File 1.

Eligible studies were original human studies reporting ECV estimation using SECT, DECT, or PCCT. PCCT studies included early clinical human investigations on available PCCT platforms; no phantom-only or preclinical studies were eligible. Studies with any comparator (including CMR or clinical reference standards) and both prospective and retrospective designs were eligible. Reviews, editorials, letters without original data, case reports or case series, animal or *in vitro* studies, phantom studies, and duplicate or overlapping cohorts were excluded. Duplicates were removed before title and abstract screening. Screening and full-text assessment were independently performed by two reviewers, with disagreements resolved by consensus or consultation with a third reviewer.

Structured data extraction of technical parameters was performed when available, including CT technology (SECT, DECT, or PCCT), acquisition approach, timing of delayed imaging, contrast administration, hematocrit estimation (measured or synthetic), region-of-interest methodology, type of comparator, and reporting and definition of ECV cutoff values. These variables are systematically summarized in Supplementary Table S1. Contrast administration protocols are reported as described in the original studies and may include dose-based (mgI/kg), volume-based (mL), or rate-based (mgI/kg/s) approaches. Owing to substantial heterogeneity in CT technology, acquisition protocols, timing of delayed imaging, contrast administration, hematocrit estimation, and region-of-interest methodology, a quantitative meta-analysis was not considered appropriate. Accordingly, results were synthesized using a structured approach. Study quality was assessed using the Newcastle–Ottawa Scale (NOS).

## Results

### Study selection

The literature search across PubMed, Embase, and Scopus identified 624 records (232 from PubMed, 215 from Scopus, and 177 from Embase). After removal of 320 duplicates, 304 unique records remained for title and abstract screening. Of these, 130 articles were considered potentially relevant and underwent full-text assessment. Following application of predefined eligibility criteria, 113 studies were included in the final qualitative synthesis.

**Fig. 3:**
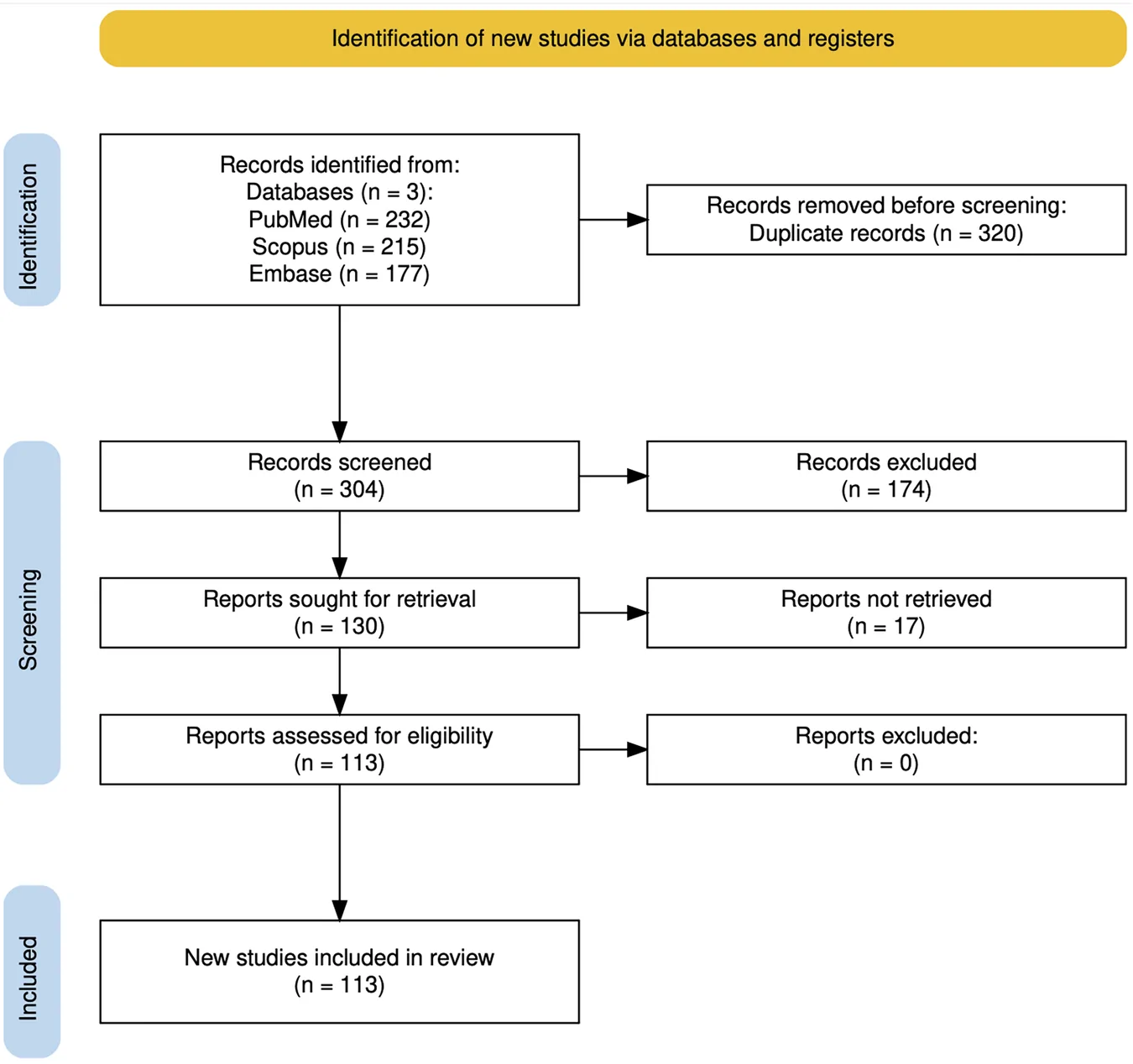
PRISMA flow diagram

Seventeen articles were excluded at the full-text stage. Reasons for exclusion included publication in a language other than English, restricted access or withdrawn articles, and lack of relevance to myocardial ECV quantification by CT. The study selection process is summarized in Fig. 3. A complete list of all included studies is provided in Supplementary Materials File 2.

### Temporal trend

The temporal trend of included studies reflects the predefined review period spanning from January 1, 2015, to December 9, 2025. A marked increase in publications was observed in recent years, with the majority of studies published between 2020 and 2025. Specifically, 12 studies (10.6%) were published in 2020, 11 (9.7%) in 2021, 14 (12.4%) in 2022, 11 (9.7%) in 2023, 21 (18.6%) in 2024, and 30 (26.5%) in 2025. This trend highlights a rapid and sustained growth of interest in CT-ECV, particularly in the last 2 years of the review period.

**Fig. 4:**
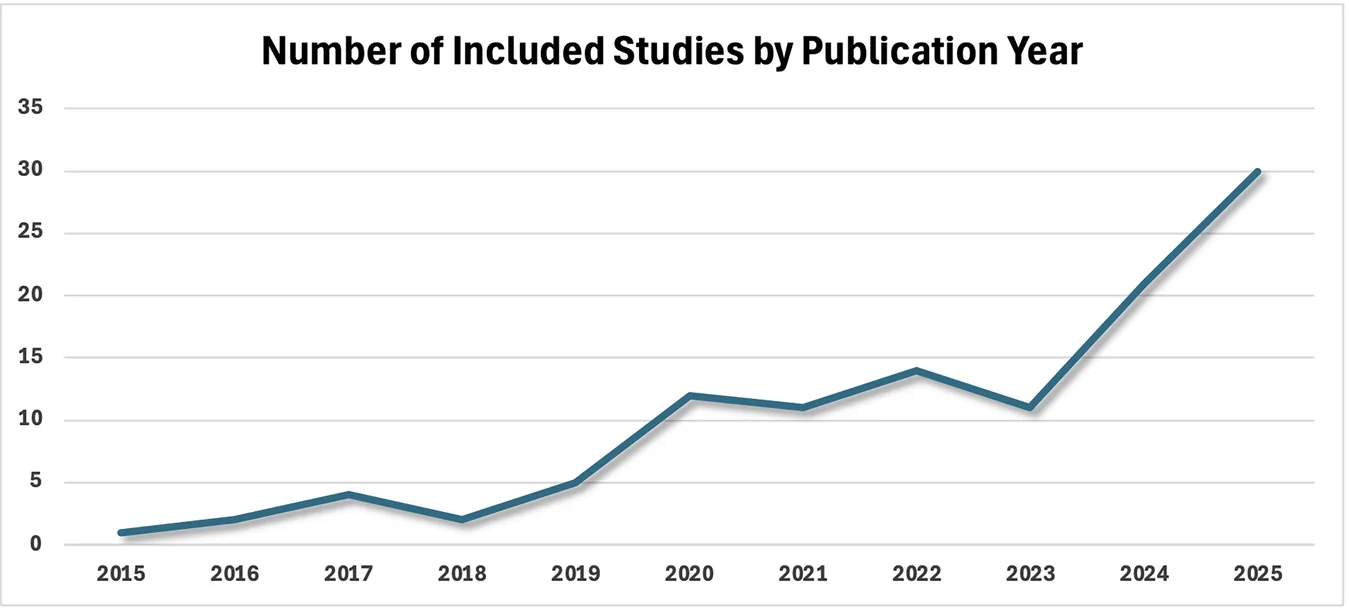
Number of included studies by publication year

**Fig. 5:**
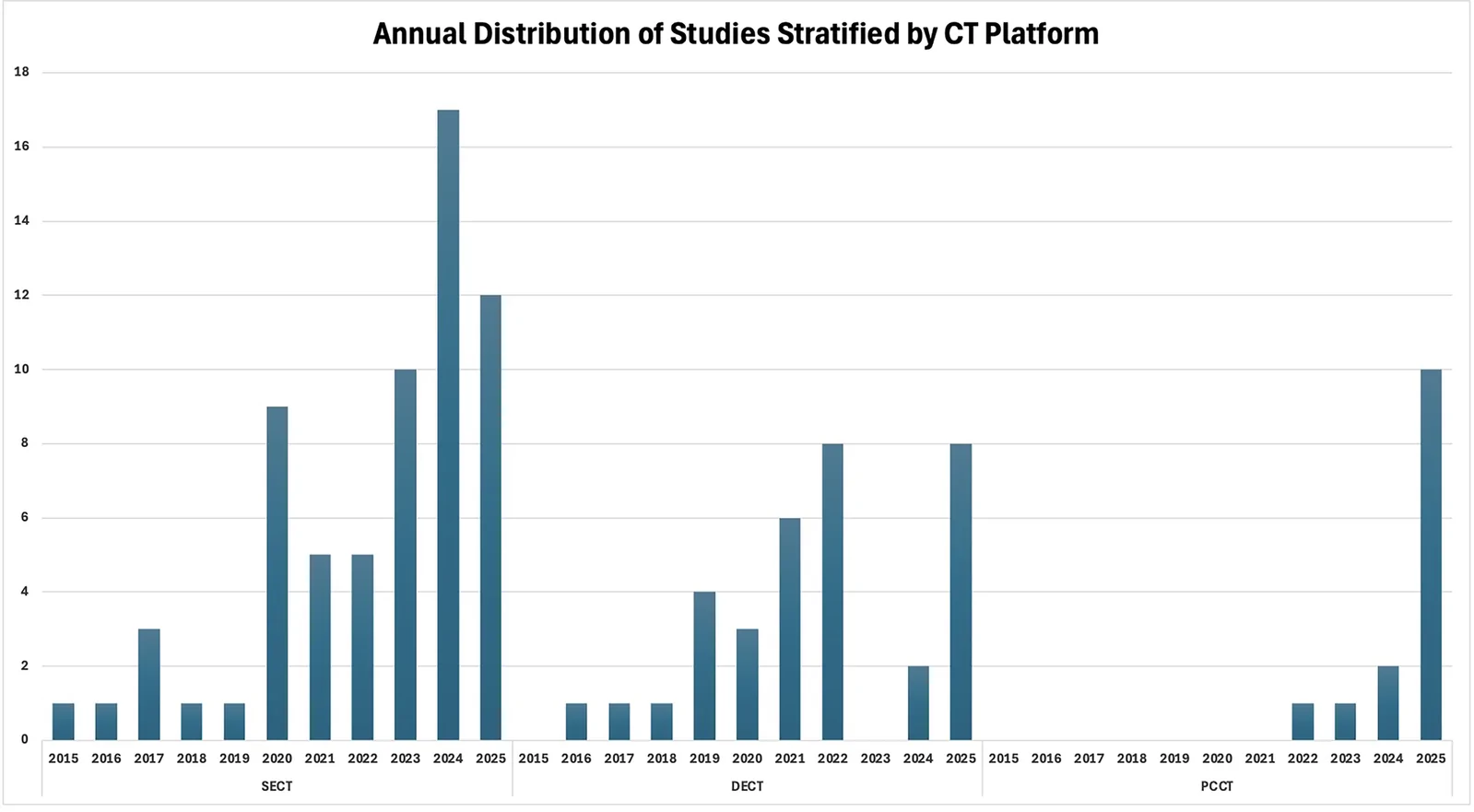
Annual distribution of studies stratified by CT platform

**Fig. 6:**
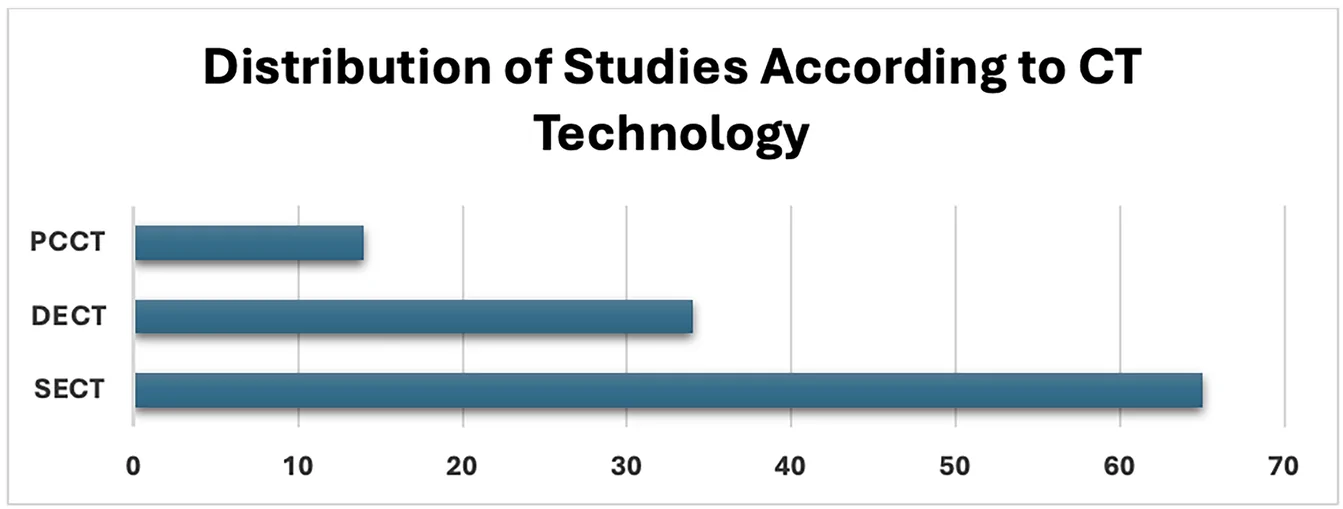
Distribution of studies according to CT technology

When stratified by CT technology, a similar temporal pattern was observed across SECT and DECT, with increasing adoption over time. Notably, studies investigating PCCT were predominantly concentrated in the most recent years, with the majority (*n* = 10) published in 2025 alone. This finding underscores the emerging role of PCCT as a key focus of contemporary research in myocardial ECV quantification. The temporal distribution of studies is illustrated in Fig. 4, with technology-specific trends shown in Figs. 5 and 6.

### Clinical settings and disease spectrum

Studies were grouped into eight main categories based on their primary clinical context. Two studies investigated both aortic stenosis and cardiac amyloidosis within the same cohorts and were therefore considered as overlapping between categories, without defining an additional category. Twenty-one studies (18.6%) investigated aortic stenosis. Fourteen studies (12.4%) focused on cardiac amyloidosis. Seven studies (6.2%) investigated heart failure, including both preserved and reduced ejection fraction phenotypes. Fourteen studies (12.4%) examined cardiomyopathies, including both ischemic and non-ischemic forms. Three studies (2.7%) evaluated myocarditis or inflammatory cardiac conditions. Thirteen studies (11.5%) were conducted in the context of cardio-oncology. Thirty-three studies (29.2%) addressed miscellaneous cardiac diseases, while eight studies (7.1%) investigated systemic diseases with secondary cardiac involvement. The overall distribution of studies across these categories is presented in Supplementary Table S2.

### Relationship between pathology and CT technology

**Fig. 7:**
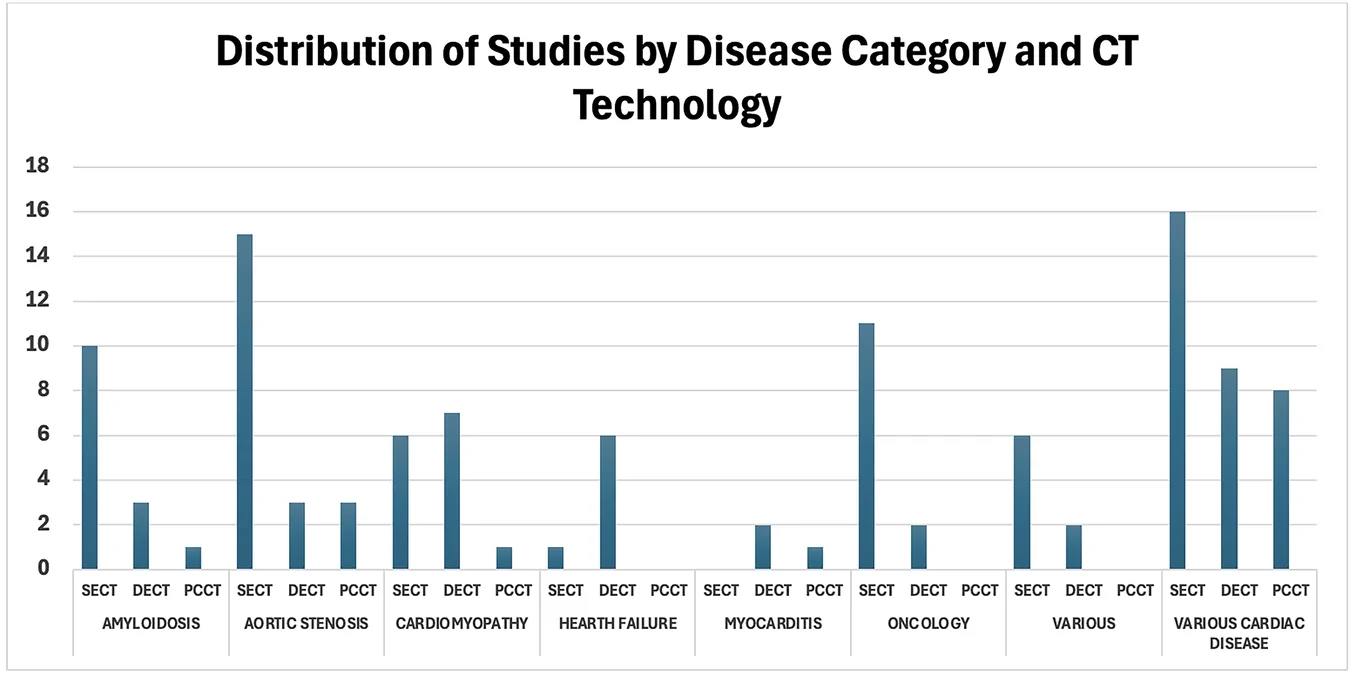
Distribution of studies by disease category and CT technology

When analyzing the relationship between clinical setting and CT technology, PCCT was most frequently used in studies classified as miscellaneous cardiac diseases. Aortic stenosis represented the second most common application of PCCT. In contrast, SECT and DECT studies were more evenly distributed across disease categories, reflecting their longer availability and integration into routine cardiac imaging. The distribution of pathologies according to CT technology is shown in Fig. 7.

### Study design

Of the 113 included studies, 67 were retrospective and 46 were prospective. The average sample size across included studies was 99.7 ± 151.3 participants (mean ± standard deviation), reflecting the predominance of small- to medium-sized cohorts in the current literature (as shown in Supplementary Fig. S1). Retrospective designs predominated in earlier studies and often relied on previously acquired CT datasets reanalyzed for myocardial ECV estimation. Prospective studies became more common in recent years, particularly in investigations using DECT and PCCT. No assessment of CT-ECV in the context of randomized controlled trials was identified.

### Reporting of ECV cutoff values

**Fig. 8:**
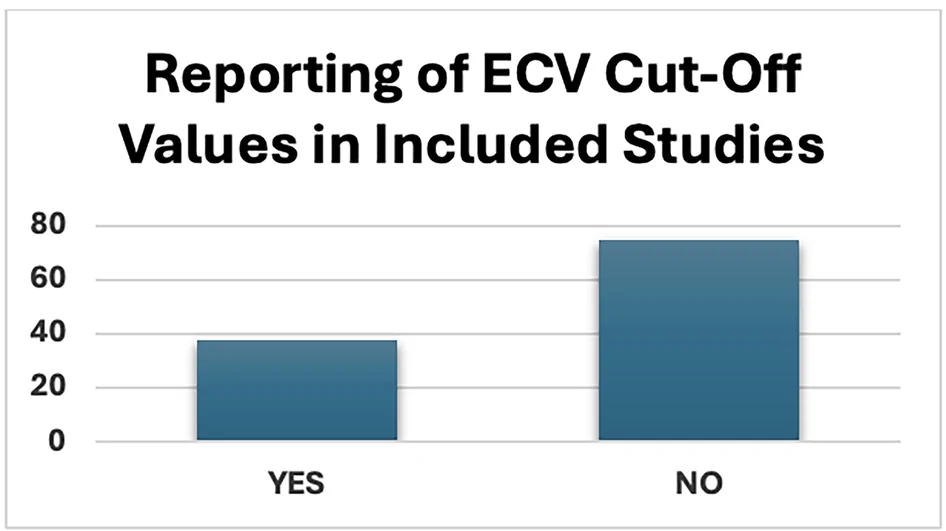
Reporting of ECV cutoff values in included studies

**Fig. 9:**
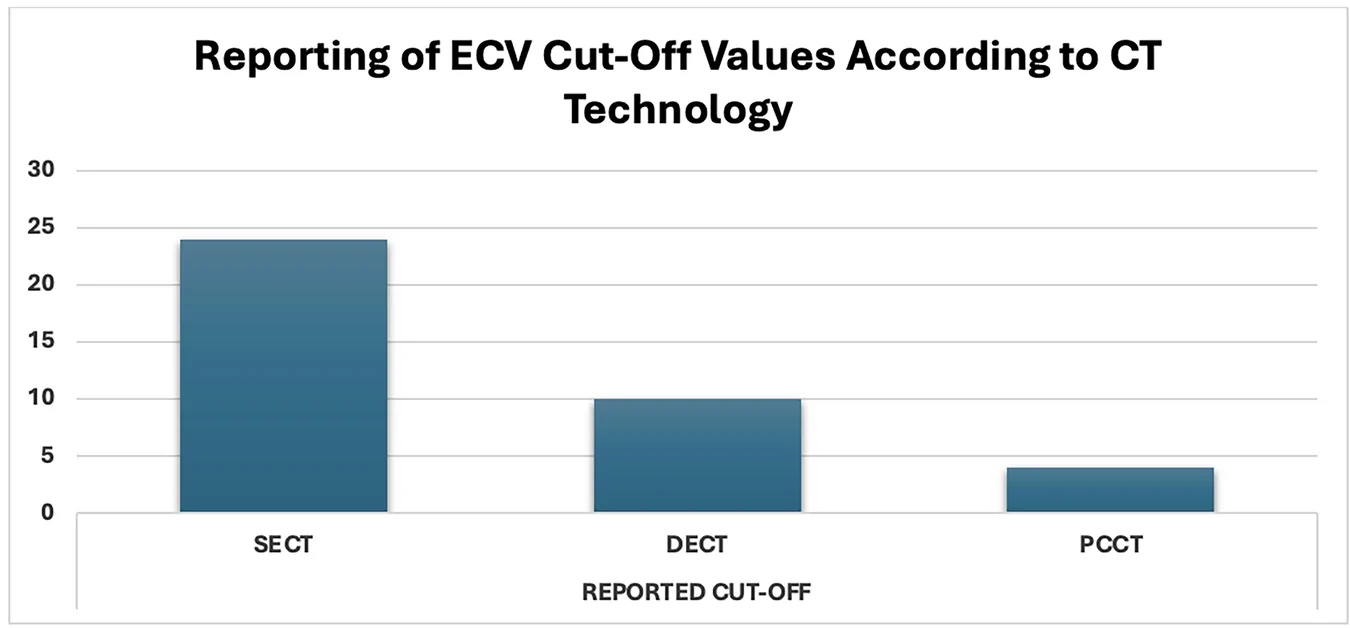
Reporting of ECV cutoff values according to CT technology

Thirty-eight studies (33.6%) reported predefined or study-derived ECV cutoff values, whereas 75 (66.4%) did not define specific thresholds. Given the substantial heterogeneity in CT technology, acquisition protocols, and study populations, these cutoff values were not pooled or quantitatively analyzed. Other studies, particularly those focused on methodological validation or technical feasibility, reported continuous ECV measurements without defining explicit thresholds. This approach was particularly common in early PCCT investigations. The distribution of studies reporting ECV cutoff values is summarized in Fig. 8, while their distribution according to CT technology is shown in Fig. 9.

### Quality assessment

Study quality was assessed using the NOS. Scores ranged from 3 to 9, with a mean value of 6.3. Most studies achieved moderate quality ratings. Common limitations included potential selection bias, limited comparability between study groups, and variability in outcome assessment or imaging analysis methods. Because the NOS is not specifically designed for diagnostic accuracy or imaging studies, scores were interpreted descriptively rather than as criteria for inclusion or exclusion.

### Integrated descriptive synthesis

Overall, the results demonstrate a rapid expansion of research on CT-ECV. The field has evolved technologically from SECT to DECT and more recently to PCCT, reflecting broader developments in cardiac CT. Studies were conducted across a wide spectrum of cardiac and systemic diseases, supporting CT-ECV as a general marker of myocardial interstitial expansion. Although retrospective studies remain common, the growing number of prospective investigations indicates increasing methodological maturity. Variability in study design, CT methodology, and ECV cutoff reporting highlights the need for greater standardization in future research.

## Discussion

Over the last decade, interest in CT-ECV has increased rapidly. The marked increase in publications observed after 2023 in this review further supports the concept of CT-ECV as a rapidly evolving field, driven by both technological advancements and growing clinical interest in quantitative myocardial tissue characterization.

The maturity of evidence varies across disease categories. More robust and consistent evidence is available for aortic stenosis and cardiac amyloidosis, where CT-ECV has been investigated in multiple cohorts with concordant findings [6–16]. Intermediate evidence exists for heart failure, cardiomyopathies, and cardio-oncology, where data are increasing but remain heterogeneous in methodology and clinical endpoints [17–34]. In contrast, other areas, including myocarditis, systemic diseases, and heterogeneous cardiac conditions, remain in earlier exploratory phases, often characterized by smaller cohorts and primarily feasibility-oriented studies [16, 35–61].

Initially explored as a methodological feasibility topic, CT-ECV has progressively expanded into a broad clinical landscape encompassing multiple cardiac and systemic diseases characterized by diffuse myocardial interstitial involvement. Importantly, reported ECV cutoff values should be interpreted with caution. In most studies, thresholds were either derived within individual cohorts [6, 8, 17, 21, 26, 62] or proposed in specific disease contexts [12, 13, 25, 37, 57, 63–66], limiting their generalizability. Furthermore, differences in CT technology, acquisition protocols, timing of delayed imaging, hematocrit estimation, and region-of-interest definition may directly influence ECV measurements, further hindering cross-study comparability. As a result, currently available cutoff values cannot be considered universally applicable across different clinical settings or imaging platforms.

**Fig. 10:**
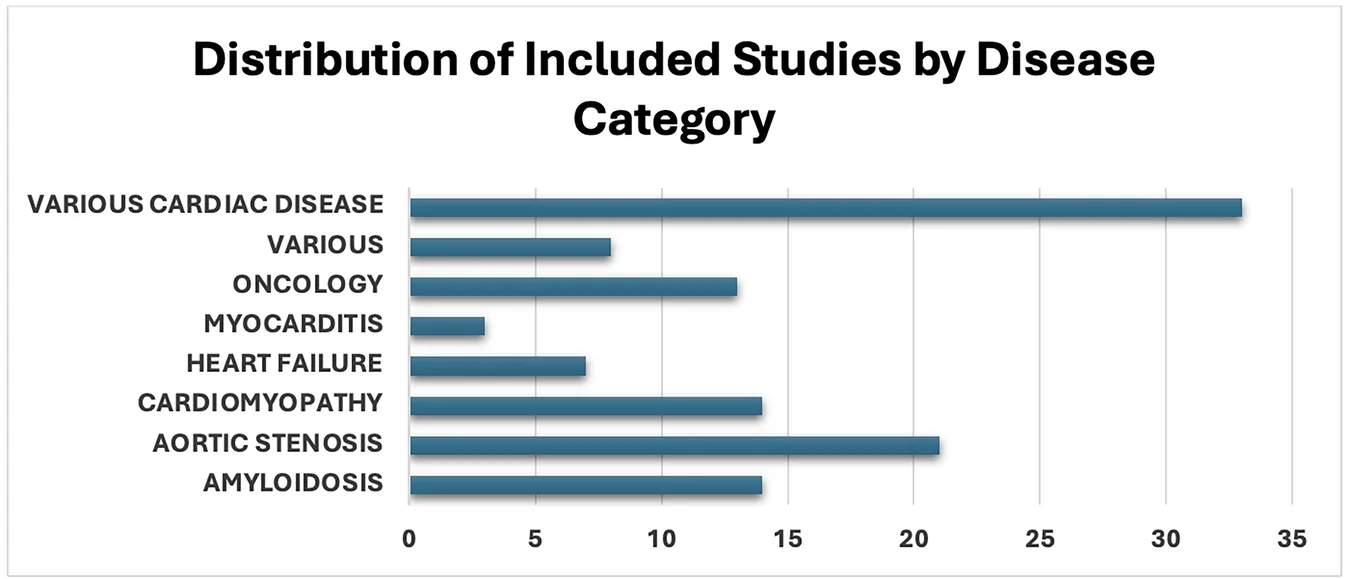
Distribution of included studies by disease category

The evolution from SECT to DECT and, more recently, to PCCT reflects a broader technological transition in cardiac imaging, with progressive improvements in spectral capabilities and quantitative accuracy. However, this rapid technological development has also contributed to methodological heterogeneity across studies. Disease-specific applications, beginning with aortic stenosis, provide a useful framework to interpret the current evidence (Fig. 10).

### Aortic stenosis

Aortic stenosis represents the most extensively investigated clinical setting for CT-ECV, reflecting the central role of diffuse interstitial fibrosis in pressure overload–related remodeling. Across multiple cohorts, ECV increased with disease severity and identified maladaptive myocardial changes preceding overt systolic dysfunction [6–10, 67, 68]. Integration of delayed ECV acquisition into routine transcatheter aortic valve implantation–TAVI planning CT enables simultaneous evaluation of valvular anatomy, vascular access, and myocardial tissue characteristics without additional imaging [7, 9, 68]. Elevated ECV was consistently associated with mortality, heart failure hospitalization, impaired reverse remodeling, and procedural vulnerability, while lower ECV correlated with improved post-intervention recovery [6]. Associations with extravalvular cardiac damage, frailty, and systemic inflammation further support ECV as an integrative marker of disease burden [10, 69, 70]. Dedicated studies also demonstrated that baseline ECV predicts adverse outcomes and reduced functional recovery following transcatheter valve implantation [71]. From a methodological perspective, dual-energy CT improved robustness through iodine-based quantification [7–9], while recent photon-counting CT investigations demonstrated high feasibility and integration with functional parameters such as myocardial strain [62, 63, 72]. CT-ECV has also shown value in identifying concomitant transthyretin amyloidosis in transcatheter aortic valve implantation candidates, enabling myocardial phenotyping beyond conventional imaging markers [64, 73].

### Cardiac amyloidosis

Cardiac amyloidosis is another setting in which CT-ECV has demonstrated consistent diagnostic and prognostic value. Early validation studies reported strong agreement between CT-ECV, CMR-ECV, and histological amyloid burden, enabling reliable discrimination from other causes of myocardial hypertrophy [11, 74]. Subsequent studies confirmed markedly elevated ECV values in both light-chain and transthyretin amyloidosis, correlating with ventricular remodeling, biomarkers, and functional impairment [12, 65]. Increased ECV was independently associated with adverse outcomes, particularly in transthyretin amyloidosis, supporting its role as a quantitative marker of disease burden. Opportunistic ECV assessment during routine CT imaging has also enabled identification of previously unrecognized amyloidosis in selected populations [13, 14]. Both SECT and DECT approaches demonstrated good reproducibility, while DECT enabled iodine-based ECV estimation from delayed acquisitions alone [11, 74]. Emerging photon-counting CT studies showed excellent agreement with CMR and improved spectral performance, facilitating simultaneous evaluation of coronary disease and myocardial infiltration in this complex population [15, 16]. The overlap between amyloidosis and aortic stenosis further highlights the value of CT-ECV in integrated myocardial phenotyping [64].

### Heart failure

Diffuse interstitial fibrosis is a central substrate of adverse myocardial remodeling in heart failure. CT-ECV has demonstrated strong agreement with CMR measurements, particularly when iodine-based DECT techniques are used. Evidence is most mature in heart failure with preserved ejection fraction, where elevated ECV correlated with disease severity and adverse clinical outcomes [17, 18]. In broader heart failure populations, including non-ischemic phenotypes, ECV varied across ejection fraction categories and was associated with major adverse cardiovascular events, supporting its potential role in risk stratification beyond conventional functional parameters [19].

### Cardiomyopathies

CT-ECV has been investigated across a wide spectrum of cardiomyopathies, including both ischemic and non-ischemic phenotypes. In ischemic cardiomyopathy, CT-ECV enables quantification of diffuse interstitial fibrosis in non-infarcted myocardium, complementing focal scar assessment and providing information beyond conventional late iodine enhancement [20]. Dual-energy CT iodine mapping demonstrated good agreement with cardiovascular magnetic resonance and histological references, enabling differentiation between scarred and remote myocardium and supporting reproducible global and segmental ECV measurements. In non-ischemic cardiomyopathies, including dilated and hypertrophic phenotypes, CT-ECV consistently reflected diffuse myocardial remodeling and fibrosis burden. Studies using both SECT and DECT demonstrated significantly elevated ECV values compared with healthy myocardium and correlations with ventricular dysfunction, chamber dilation, and adverse clinical profiles [21–23]. In dilated cardiomyopathy, CT-ECV showed prognostic value, independently predicting major adverse cardiac events, heart failure hospitalization, and arrhythmic outcomes [20, 23]. Whole-heart CT acquisition ensured high myocardial segment evaluability even in patients with advanced ventricular remodeling or contraindications to CMR.

Methodological investigations have explored optimization and standardization of ECV quantification. Comparative analyses of subtraction-based and iodine-density approaches demonstrated good consistency, with iodine-based techniques showing improved reproducibility and reduced sensitivity to misregistration. Automated postprocessing strategies have also been proposed to facilitate scalable clinical implementation. In a large DECT cohort including both ischemic and non-ischemic cardiomyopathies, a fully automated ECV estimation algorithm achieved excellent diagnostic performance while substantially reducing postprocessing time [24]. Early experiences with photon-counting CT suggest additional advantages, including improved spectral separation and reduced noise, enabling more robust iodine quantification in complex myocardial phenotypes [25].

### Myocarditis and inflammatory cardiac conditions

In acute myocarditis, myocardial ECV reflects diffuse inflammatory burden. DECT studies demonstrated significantly elevated ECV compared with controls and correlations with biomarkers of myocardial injury and adverse events [35, 36]. PCCT further improved diagnostic performance and enabled combined coronary evaluation and myocardial tissue characterization within a single examination [37, 38].

### Cardio-oncology

In cardio-oncology, CT-ECV has emerged as a sensitive marker of subclinical myocardial injury related to cancer therapies. Multiple studies demonstrated early ECV increases following anthracycline therapy or thoracic radiotherapy, often preceding measurable functional decline [26, 28, 75]. Longitudinal studies linked ECV progression to cumulative treatment exposure and adverse cardiovascular outcomes, including in patients receiving androgen deprivation therapy [29–32]. Advanced CT technologies, including DECT and PCCT, enabled integration of myocardial ECV assessment into routine oncologic imaging workflows [30, 33, 34, 66].

### Various cardiac diseases

A substantial body of literature has investigated CT-ECV in heterogeneous cardiac populations, reflecting its role as a disease-agnostic biomarker of myocardial interstitial remodeling. These studies encompassed diverse clinical conditions, including hypertrophic cardiomyopathy, atrial fibrillation, spontaneous coronary artery dissection, ventricular arrhythmias, coronary microvascular dysfunction, microvascular ischemia, and valvular diseases such as aortic regurgitation. Across mixed cohorts, CT-ECV demonstrated sensitivity to diffuse remodeling and good agreement with CMR [39–42]. Methodological studies explored optimization of acquisition timing and reconstruction, particularly using spectral CT platforms [43–47, 76]. Early PCCT investigations demonstrated robust iodine-based ECV estimation across diverse clinical scenarios [49–53, 77], while exploratory analyses suggested associations with adverse remodeling and cardiovascular risk [54]. Additional applications included peri-procedural assessment and longitudinal monitoring [55].

### Various systemic diseases with cardiac involvement

CT-ECV has also been investigated in systemic diseases with secondary cardiac involvement, including COVID-19, HIV infection, end-stage renal disease, pulmonary hypertension, diabetes mellitus, and coronary microvascular dysfunction. Across these conditions, elevated ECV reflected diffuse myocardial injury driven by systemic inflammation or metabolic dysregulation [56–60, 78]. In end-stage renal disease, ECV demonstrated independent prognostic value and provided clinically relevant information in settings where gadolinium-enhanced CMR is contraindicated [57, 61]. CT-ECV has also been evaluated in acute systemic conditions such as ischemic stroke, where delayed spectral CT performed after cerebrovascular CT angiography identified incidental myocardial involvement and provided prognostically relevant cardiovascular information without altering acute stroke workflows [55–61, 78].

### CT technologies for myocardial ECV assessment

The expansion of clinical applications has paralleled rapid technological evolution. Early SECT-based approaches demonstrated feasibility but were sensitive to noise and misregistration. DECT represented a major advance by enabling iodine-based material decomposition and more robust ECV quantification. PCCT represents a promising emerging technology with potential advantages in spectral performance and spatial resolution. Recent evidence also suggests improved reproducibility of quantitative myocardial imaging with photon-counting CT [79]. However, current evidence remains limited and is largely based on feasibility and early clinical studies. Further validation in larger and standardized cohorts is required before its incremental value over established CT technologies can be fully established [80]. The present review demonstrates a clear recent increase in PCCT-based ECV studies, reflecting a shift toward next-generation CT platforms. To our knowledge, this is the first systematic review mapping CT-ECV across SECT, DECT, and PCCT technologies while contextualizing the emerging role of photon-counting CT within the technological evolution of cardiac CT.

### Limitations

The available evidence remains predominantly observational and largely derived from single-center studies, with considerable heterogeneity, precluding quantitative synthesis. In addition, the relatively small sample sizes observed across many included studies (mean sample size: 99.67 participants) may limit the statistical power and generalizability of the findings. Additional heterogeneity in acquisition protocols should also be acknowledged. Qualitative appraisal of the literature revealed substantial variability in imaging protocols used for myocardial ECV quantification [1]. Representative examples were observed in studies of aortic stenosis [70], cardio-oncology [28], and various cardiac diseases [55]. These references are cited only as illustrative examples rather than a comprehensive technical comparison. Differences in acquisition timing and contrast injection protocols may influence measured ECV values and contribute to inter-study variability. Furthermore, in some studies, the CT acquisition protocol used for ECV estimation was only partially described or insufficiently detailed [75]. Although these aspects were not systematically analyzed, they represent an important methodological issue that may currently limit cross-study comparability and hinder the establishment of universally applicable ECV reference ranges or diagnostic cutoff values. Greater harmonization of acquisition protocols and more comprehensive reporting of technical parameters will therefore be essential to support reproducibility and facilitate clinical standardization. The inclusion of only English-language studies may have introduced language bias.

### Implications for clinical practice and policy

CT-ECV has demonstrated feasibility across diverse disease settings, often within existing imaging workflows. Integration into routine cardiac and thoracic CT examinations may enhance diagnostic and prognostic yield, although broader adoption will require standardized acquisition protocols, harmonized reporting, and clearer definition of clinical use cases.

### Implications for future research

Overall, CT-ECV has emerged as a versatile imaging biomarker with potential applications across a wide spectrum of cardiac and systemic diseases. However, the heterogeneity of current evidence, particularly in terms of acquisition protocols, analysis methods, and reporting of cutoff values, highlights the need for standardization. Future research should prioritize harmonized imaging protocols and multicenter validation to facilitate the clinical translation of CT-ECV. PCCT may further enhance accuracy and enable more comprehensive myocardial tissue characterization within routine CT imaging.

## Conclusions

CT-ECV has rapidly evolved from a feasibility-oriented technique into a versatile quantitative imaging biomarker investigated across a wide spectrum of cardiac and systemic diseases. As highlighted by this systematic review, myocardial ECV assessment using CT has been applied in several clinical settings, ranging from valvular heart disease and cardiomyopathies to inflammatory, oncologic, and systemic conditions, reflecting the central role of diffuse interstitial myocardial involvement as a shared pathophysiological substrate.

A key finding emerging from the integrated discussion of disease-specific evidence is the progressive technological transition underpinning this expansion. While early applications relied on single-energy CT, the adoption of dual-energy techniques has enabled more robust iodine-based ECV quantification and improved workflow integration. More recently, photon-counting CT has emerged as a pivotal development, offering superior spectral performance, reduced image noise, and enhanced spatial resolution. The growing number of PCCT-based studies identified in this review indicates a clear shift in research focus toward next-generation CT platforms and supports their potential to further refine myocardial tissue characterization.

Despite these advances, substantial methodological heterogeneity persists across studies, including variability in acquisition protocols, postprocessing strategies, and reporting of ECV cutoff values. This heterogeneity currently limits comparability and hampers broader clinical translation. Consequently, future efforts should prioritize standardization, multicenter validation, and prospective evaluation of clinical impact across technologies and disease contexts.

Within this framework, CT-ECV holds promise as an accessible and scalable imaging biomarker that can be integrated into routine cardiac and thoracic CT workflows. As CT technology continues to evolve, harmonized approaches to myocardial ECV quantification will be essential to support its adoption as a robust tool for comprehensive cardiovascular phenotyping in both cardiac and systemic diseases.

## Supplementary information

**Additional file: Figure S1** - Observational study design distribution. **Table S1** – Data extraction. **Table S2** – Overall distribution of studies across pathologies.

Supplementary Materials File 1

Supplementary Materials File 2

## Data Availability

All data generated or analyzed during this study are included in this published article and its supplementary information files. The full search strategies for each database are provided in the Supplementary Materials.

## Abbreviations

CMR: Cardiovascular magnetic resonance
CT: Computed tomography
CT-ECV: CT-derived myocardial extracellular volume
DECT: Dual-energy CT
ECV: Cardiac extracellular volume
NOS: Newcastle–Ottawa scale
PCCT: Photon-counting CT
SECT: Single-energy CT
