# Supplementary material for: CT-derived myocardial extracellular volume: current evidence, clinical utility, and the emerging role of photon-counting CT—a systematic review
Source: Eur Radiol Exp. 2026 Sep 22;10:142. doi: 10.1186/s41747-026-00808-w (PMC13598009; doi:10.1186/s41747-026-00808-w)
Supplement: Supplementary file 1 [file 41747_2026_808_MOESM1_ESM.pdf]

CT-derived myocardial extracellular volume: current evidence, clinical utility, and the emerging role of photon-counting CT. A systematic review

ELECTRONIC SUPPLEMENTARY MATERIAL

Search strings

PubMed

(("extracellular volume" OR "ECV" OR "extracellular volume fraction") AND ("heart" OR "myocardium" OR "cardiac") AND ("computed tomography" OR "CT" OR "dual-energy CT" OR "DECT" OR "cardiac CT" OR "photon-counting CT" OR "photon counting CT" OR "photon-counting detector")) AND ("2015/01/01"[Date - Publication]: "3000" [Date - Publication]) NOT ("animals"[Title/Abstract] OR "in vitro"[Title/Abstract] OR "review"[Publication Type])

Scopus

TITLE-ABS ("extracellular volume" OR "extracellular volume fraction" OR ECV) AND TITLE-ABS ("heart" OR myocard* OR cardiac) AND TITLE-ABS ("computed tomography" OR "cardiac CT" OR "dual-energy CT" OR "dual energy CT" OR DECT OR "photon-counting CT" OR "photon counting CT" OR "photon-counting detector") AND NOT TITLE-ABS ("in vitro") AND NOT DOCTYPE(review)

Embase

('extracellular volume':ab,ti OR 'extracellular volume fraction':ab,ti OR ecv:ab,ti) AND ('heart':ab,ti OR myocard*:ab,ti OR cardiac:ab,ti) AND ('computed tomography':ab,ti OR 'cardiac ct':ab,ti OR 'dual-energy ct':ab,ti OR 'dual energy ct':ab,ti OR dect:ab,ti OR 'photon-counting ct':ab,ti OR 'photon counting ct':ab,ti OR 'photon-counting detector':ab,ti) NOT ('in vitro study'/exp) NOT 'review'/it NOT 'conference abstract'/it

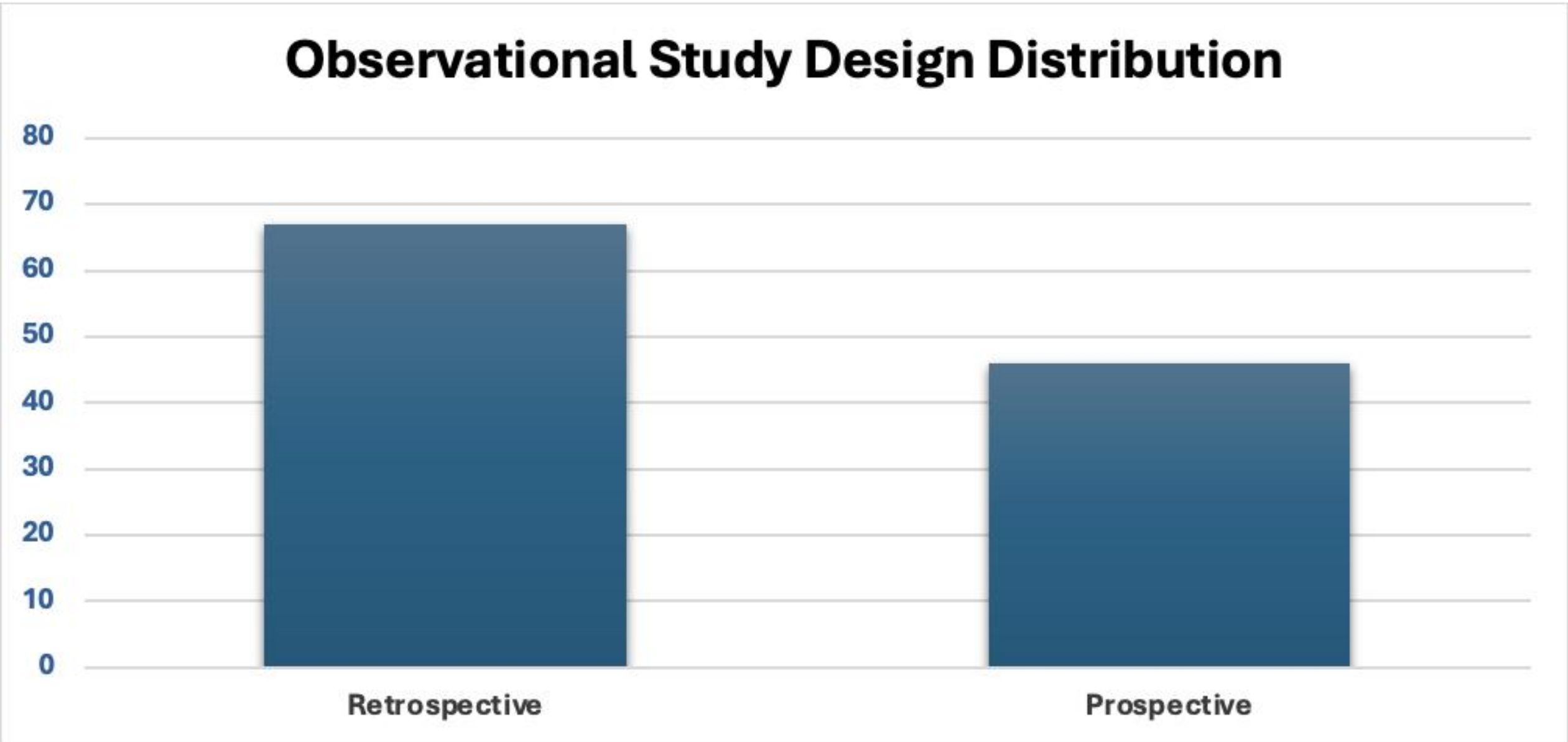

Figure S1 - Observational study design distribution

FULL LIST OF INCLUDED ARTICLES[1–113]

1. Monti CB, Zanardo M, Bosetti T et al (2020) Assessment of myocardial extracellular volume on body computed tomography in breast cancer patients treated with anthracyclines. Quant Imaging Med Surg 10:934–944. https://doi.org/10.21037/qims.2020.04.05

2. Yashima S, Takaoka H, Ota J et al (2024) Extracellular volume by computed tomography is useful for prediction of prognosis in dilated cardiomyopathy cases with heart failure with reduced ejection fraction. J Cardiovasc Dev Dis 11:399. https://doi.org/10.3390/jcdd11120399

3. Borrazzo C, d'Ettorre G, Ceccarelli G et al (2020) Unexpected increase of myocardial extracellular volume fraction in low cardiovascular risk hiv patients. Transl Med Commun 5:24. https://doi.org/10.1186/s41231-020-00077-8

4. Aoki S, Takaoka H, Kanaeda T et al (2025) Extracellular volume fraction analysis on cardiac computed tomography is useful for predicting the prognosis of hypertrophic cardiomyopathy. J Cardiovasc Dev Dis 12:372. https://doi.org/10.3390/jcdd12090372

5. Yu Y, Yang W, Dai X et al (2023) Microvascular myocardial ischemia in patients with diabetes without obstructive coronary stenosis and its association with angina. Korean J Radiol 24:1081–1092. https://doi.org/10.3348/kjr.2023.0297

6. van Assen M, De Cecco CN, Sahbaee P et al (2019) Feasibility of extracellular volume quantification using dual-energy ct. J Cardiovasc Comput Tomogr 13:81–84. https://doi.org/10.1016/j.jcct.2018.10.011

7. Wu H, Huang Y, Yang M et al (2024) Quantitative assessment of early changes in myocardial extracellular volume during postoperative adjuvant chemotherapy in patients with breast cancer via dual-layer spectral detector computed tomography: a cohort study. Quant Imaging Med Surg 15:404–416. https://doi.org/10.21037/qims-24-777

8. Lisi C, Mergen V, Moser LJ et al (2025) Relationship between myocardial strain and extracellular volume: exploratory study in patients with severe aortic stenosis undergoing photon-counting detector ct. Diagnostics 15:224. https://doi.org/10.3390/diagnostics15020224

9. Martuszewski A, Paluszkiewicz P, Poręba R, Gać P (2025) Increased left ventricular myocardial extracellular volume assessed by cardiac computed tomography as a consequence of aortic stenosis and coexisting cardiovascular risk factors. J Clin Med 14:4435. https://doi.org/10.3390/jcm14134435

10. Capra D, Monti CB, Luporini AG et al (2020) Computed tomography-derived myocardial extracellular volume: an early biomarker of cardiotoxicity in esophageal cancer patients undergoing radiation therapy. Insights Imaging 11:120. https://doi.org/10.1186/s13244-020-00922-2

11. Kidoh M, Oda S, Tabata N et al (2025) CT-derived extracellular volume fraction in aortic stenosis, cardiac amyloidosis, and dual pathology. Eur Heart J Cardiovasc Imaging 26:509–517. https://doi.org/10.1093/ehjci/jeae320

12. Qi R-X, Jiang J-S, Shao J et al (2022) Measurement of myocardial extracellular volume fraction in patients with heart failure with preserved ejection fraction using dual-energy computed tomography. Eur Radiol 32:4253–4263. https://doi.org/10.1007/s00330-021-08514-4

13. Baggiano A, Conte E, Spiritigliozzi L et al (2023) Quantification of extracellular volume with cardiac computed tomography in patients with dilated cardiomyopathy. J Cardiovasc Comput Tomogr 17:261–268. https://doi.org/10.1016/j.jcct.2023.04.005

14. McShane JN, Tavoosi A, El Mais H et al (2025) CT derived ecv in severe aortic stenosis: prognosticator and screening test for co-existent transthyretin cardiac amyloidosis. American Heart Journal Plus: Cardiology Research and Practice 56:100575. https://doi.org/10.1016/j.ahjo.2025.100575

15. Mergen V, Reiner MF, Klambauer K et al (2025) Myocardial characterization using late enhancement photon-counting detector ct in ventricular arrhythmia: comparison with electroanatomical mapping. Insights Imaging 16:187. https://doi.org/10.1186/s13244-025-02069-4

16. Gkizas C, Longere B, Sliwicka O et al (2025) Photon-counting ct-derived extracellular volume in acute myocarditis: comparison with cardiac mri. Diagn Interv Imaging 106:255–263. https://doi.org/10.1016/j.diii.2025.03.001

17. Deng J, Wang Y, Qi T et al (2025) Myocardial extracellular volume fraction with spectral detector computed tomography for risk stratification in non-ischemic heart failure. Radiol Med 130:1092–1104. https://doi.org/10.1007/s11547-025-02002-1

18. Yamaguchi S, Oda S, Kidoh M et al (2025) Imaging features of wild-type transthyretin amyloid cardiomyopathy in gated and non-gated contrast-enhanced ct. Eur Radiol 35:7064–7073. https://doi.org/10.1007/s00330-025-11631-z

19. Ohta Y, Kitao S, Watanabe T et al (2017) Measurement of myocardial extracellular volume fraction from iodine density images using single-source, dual-energy computed tomography: a feasibility study. J Comput Assist Tomogr 41:750–756. https://doi.org/10.1097/RCT.0000000000000587

20. Nishikawa Y, Takaoka H, Kanaeda T et al (2023) A new composite indicator consisting of left ventricular extracellular volume, n-terminal fragment of b-type natriuretic peptide, and left ventricular end-diastolic volume is useful for predicting reverse remodeling after catheter ablation for atrial fibrillation. Heart Vessels 38:721–730. https://doi.org/10.1007/s00380-022-02220-x

21. Saberwal B, Patel K, Klotz E et al (2025) Myocardial extracellular volume fraction by computed tomography vs. cardiovascular magnetic resonance imaging in patients with stable chest pain. European Heart Journal - Imaging Methods and Practice 3:qyaf019. https://doi.org/10.1093/ehjimp/qyaf019

22. Koike H, Cheng VY, Lesser A et al (2023) Importance of imaging-acquisition protocol and post-processing analysis for extracellular volume fraction assessment by computed tomography. J Cardiovasc Comput Tomogr 17:222–225. https://doi.org/10.1016/j.jcct.2023.02.007

23. Kokawa T, Nakamura S, Takafuji M, Yamazaki A, Sakuma H, Kitagawa K (2025) Impact of contrast medium volume on myocardial extracellular volume measurement accuracy in first-generation photon-counting detector ct. Int J Cardiovasc Imaging 42:423–434. https://doi.org/10.1007/s10554-025-03437-3

24. Hayashi H, Oda S, Kidoh M et al (2024) Myocardial extracellular volume quantification in cardiac amyloidosis: a comparative study between cardiac computed tomography and magnetic resonance imaging. Eur Radiol 34:1016–1025. https://doi.org/10.1007/s00330-023-10129-w

25. Klambauer K, Klotz E, Moser LJ et al (2026) Myocardial late enhancement with photon-counting detector ct in spontaneous coronary artery dissection: prospective comparison with cardiac mri. Invest Radiol 61:32–40. https://doi.org/10.1097/RLI.0000000000001203

26. Abadia AF, Aquino GJ, Schoepf UJ et al (2022) Automated dual-energy computed tomography-based extracellular volume estimation for myocardial characterization in patients with ischemic and nonischemic cardiomyopathy. J Thorac Imaging 37:307–314. https://doi.org/10.1097/RTI.0000000000000656

27. Kokawa T, Kitagawa K, Nakamura S, Takafuji M, Oya T, Sakuma H (2025) Myocardial late enhancement using dual-source ct: intraindividual comparison of single-energy shuttle and dual-energy acquisition. Insights Imaging 16:64. https://doi.org/10.1186/s13244-025-01944-4

28. Shao J, Jiang J-S, Wang X-Y et al (2022) Measurement of myocardial extracellular volume using cardiac dual-energy computed tomography in patients with ischaemic cardiomyopathy: a comparison of different methods. Int J Cardiovasc Imaging 38:1591–1600. https://doi.org/10.1007/s10554-022-02532-z

29. Yuan J, Ding X, Yang W et al (2025) The impact of lipoprotein(a) level on cardiac pathologies in diabetes: a cardiac ct study. Eur Radiol 35:220–231. https://doi.org/10.1007/s00330-024-10903-4

30. Gkizas C, Longere B, Bechrouri S et al (2025) Photon-counting ct myocardial extracellular volume: a non-invasive biomarker for fibrosis in patients with hypertrophic cardiomyopathy. Diagn Interv Imaging 107:17–24. https://doi.org/10.1016/j.diii.2025.09.001

31. Belmonte M, Paolisso P, de Oliveira EK et al (2025) Association of cardiac damage and computed tomography derived extracellular volume in patients undergoing transcatheter aortic valve implantation. Canadian Journal of Cardiology 41:1546–1557. https://doi.org/10.1016/j.cjca.2025.02.031

32. Rodriguez-Granillo GA, Cirio JJ, Ciardi C et al (2022) Hyperacute incidental late myocardial enhancement in ischemic stroke using chest spectral ct: relationship with etiology. Rev Cardiovasc Med 23:93. https://doi.org/10.31083/j.rcm2303093

33. Kojima T, Yamasaki Y, Nishigake D et al (2025) Clinical utility of non-gated 4-min delayed dual-energy ct for myocardial extracellular volume quantification. British Journal of Radiology 98:600–606. https://doi.org/10.1093/bjr/tqaf022

34. Lorenzatti DA, Filtz A, Piña P et al (2025) Comparison of dual-energy iodine and standard subtraction methods for myocardial extracellular volume quantification using cardiac computed tomography. J Cardiovasc Comput Tomogr 19:483–487. https://doi.org/10.1016/j.jcct.2025.03.009

35. Shiyovich A, Plakht Y, Hammer Y et al (2022) Relation of serum albumin levels to myocardial extracellular volume in patients with severe aortic stenosis. American Journal of Cardiology 163:71–76. https://doi.org/10.1016/j.amjcard.2021.09.044

36. Bao S, Zhou M, Wang M, Xu Z, Yan F, Yang W (2025) Dual-energy based myocardial extracellular volume quantification with photon-counting detector and energy-integrating detector dual-source computed tomography: comparison with cardiac magnetic resonance. Eur Radiol 36:1989–2000. https://doi.org/10.1007/s00330-025-11942-1

37. Treibel TA, Fontana M, Steeden JA et al (2017) Automatic quantification of the myocardial extracellular volume by cardiac computed tomography: synthetic ecv by cct. J Cardiovasc Comput Tomogr 11:221–226. https://doi.org/https://doi.org/10.1016/j.jcct.2017.02.006

38. Popp S, Beitzke D, Strassl A et al (2025) Evaluation of extracellular volume and coronary artery disease in cardiac amyloidosis using photon-counting ct. Invest Radiol 60:698–707. https://doi.org/10.1097/RLI.0000000000001198

39. Pankaj M, Donghee H, Billy C et al (2024) Predictive value of cta-derived extracellular volume for pacemaker implantation post-tavr in low-flow low-gradient aortic stenosis. JACC Cardiovasc Imaging 17:563–565. https://doi.org/10.1016/j.jcmg.2023.11.012

40. Zhang X, Shen H, Xu Q et al (2025) CT derived myocardial extracellular volume predict major adverse cardiovascular events in patients with prostate cancer. Eur J Radiol 192:112371. https://doi.org/10.1016/j.ejrad.2025.112371

41. Chunrong T, Hesong S, Xiaoqin L et al (2024) Longitudinal evaluation of coronary arteries and myocardium in breast cancer using coronary computed tomographic angiography. JACC Cardiovasc Imaging 17:1335–1347. https://doi.org/10.1016/j.jcmg.2024.05.017

42. Sayama K, Kanaji Y, Usui E et al (2025) Increasing extracellular volume fraction on coronary cta in patients with coronary microvascular dysfunction. Circ Cardiovasc Imaging 18:e019082. https://doi.org/10.1161/CIRCIMAGING.125.018368

43. Franceschi P, Sportoletti C, Rasciti E et al (2025) Ultrafast non-ecg-gated cardiac spectral ct scanning for myocardial late iodine enhancement assessment: a feasibility study. Radiol Med 131:428–439. https://doi.org/10.1007/s11547-025-02114-8

44. Hayashi H, Oda S, Emoto T et al (2022) Myocardial extracellular volume quantification by cardiac ct in pulmonary hypertension: comparison with cardiac mri. Eur J Radiol 153:110386. https://doi.org/10.1016/j.ejrad.2022.110386

45. Hagar MT, Moore WG, Vecsey-Nagy M et al (2025) Synthetic hematocrit-based extracellular volume quantification from photon-counting ct: validation against mri and systematic bias correction. Eur J Radiol 191:112321. https://doi.org/10.1016/j.ejrad.2025.112321

46. Kagawa Y, Takafuji M, Fujita S et al (2025) Myocardial extracellular volume fraction estimations using late enhancement ct in patients with atrial fibrillation: a comparative study with cardiac mr. Int J Cardiovasc Imaging 41:419–426. https://doi.org/10.1007/s10554-024-03316-3

47. Chen ZX, Xu J, Tian W et al (2025) Dual-layer spectral computed tomography (ct) parameters for identifying severe aortic regurgitation in aortic valve disease patients. Clin Radiol 91:107067. https://doi.org/10.1016/j.crad.2025.107067

48. Klambauer K, Biondo A, Klotz E et al (2025) Photon-counting detector computed tomography compared with cardiac magnetic resonance imaging for myocardial extracellular volume quantification in spontaneous coronary artery dissection. Sci Rep 15:39923. https://doi.org/10.1038/s41598-025-23777-3

49. Yashima S, Takaoka H, Iwahana T et al (2023) Evaluation of extracellular volume by computed tomography is useful for prediction of prognosis in dilated cardiomyopathy. Heart Vessels 38:185–194. https://doi.org/10.1007/s00380-022-02154-4

50. Liu P, Lin L, Xu C et al (2022) Quantitative analysis of late iodine enhancement using dual-layer spectral detector computed tomography: comparison with magnetic resonance imaging. Quant Imaging Med Surg 12:310–320. https://doi.org/10.21037/qims-21-344

51. Kadoya Y, Omaygenc MO, Chow B, Small GR (2024) Reproducibility of myocardial extracellular volume quantification using dual-energy computed tomography in patients with cardiac amyloidosis. J Cardiovasc Comput Tomogr 19:74–80. https://doi.org/10.1016/j.jcct.2024.09.011

52. Yamada A, Kitagawa K, Nakamura S et al (2020) Quantification of extracellular volume fraction by cardiac computed tomography for noninvasive assessment of myocardial fibrosis in hemodialysis patients. Sci Rep 10:15367. https://doi.org/10.1038/s41598-020-72417-5

53. Mergen V, Sartoretti T, Klotz E et al (2022) Extracellular volume quantification with cardiac late enhancement scanning using dual-source photon-counting detector ct. Invest Radiol 57:406–411. https://doi.org/10.1097/RLI.0000000000000851

54. Aquino GJ, O'Doherty J, Schoepf UJ et al (2023) Myocardial characterization with extracellular volume mapping with a first-generation photon-counting detector ct with mri reference. Radiology 307:e222030. https://doi.org/10.1148/radiol.222030

55. Aoki S, Takaoka H, Ota J et al (2024) Strong diagnostic performance of single energy 256-row multidetector computed tomography with deep learning image reconstruction in the assessment of myocardial fibrosis. Internal Medicine 63:2499–2507. https://doi.org/10.2169/internalmedicine.2615-23

56. Hamdy A, Kitagawa K, Goto Y et al (2019) Comparison of the different imaging time points in delayed phase cardiac ct for myocardial scar assessment and extracellular volume fraction estimation in patients with old myocardial infarction. International Journal of Cardiovascular Imaging 35:917–926. https://doi.org/10.1007/s10554-018-1513-z

57. Gama F, Rosmini S, Bandula S et al (2022) Extracellular volume fraction by computed tomography predicts long-term prognosis among patients with cardiac amyloidosis. JACC Cardiovasc Imaging 15:2082–2094. https://doi.org/10.1016/j.jcmg.2022.08.006

58. Emoto T, Oda S, Kidoh M et al (2021) Myocardial extracellular volume quantification using cardiac computed tomography: a comparison of the dual-energy iodine method and the standard subtraction method. Acad Radiol 28:e119–e126. https://doi.org/10.1016/j.acra.2020.03.019

59. Egashira K, Sueta D, Tomiguchi M et al (2021) Cardiac computed tomography-derived extracellular volume fraction in late anthracycline-induced cardiotoxicity. IJC Heart and Vasculature 34:100797. https://doi.org/10.1016/j.ijcha.2021.100797

60. Koike H, Fukui M, Treibel T et al (2024) Comprehensive myocardial assessment by computed tomography: impact on short-term outcomes after transcatheter aortic valve replacement. JACC Cardiovasc Imaging 17:396–407. https://doi.org/10.1016/j.jcmg.2023.08.008

61. Si-Mohamed SA, Restier LM, Branchu A et al (2021) Diagnostic performance of extracellular volume quantified by dual-layer dual-energy ct for detection of acute myocarditis. J Clin Med 10:3286. https://doi.org/10.3390/jcm10153286

62. Ishiyama M, Kurita T, Takafuji M et al (2023) The cardiac computed tomography-derived extracellular volume fraction predicts patient outcomes and left ventricular mass reductions after transcatheter aortic valve implantation for aortic stenosis. J Cardiol 81:476–484. https://doi.org/10.1016/j.jjcc.2022.12.002

63. Deux JF, Nouri R, Tacher V et al (2021) Diagnostic value of extracellular volume quantification and myocardial perfusion analysis at ct in cardiac amyloidosis. Radiology 300:326–335. https://doi.org/10.1148/radiol.2021204192

64. Lee HJ, Im DJ, Youn JC et al (2016) Myocardial extracellular volume fraction with dual-energy equilibrium contrast-enhanced cardiac ct in nonischemic cardiomyopathy: a prospective comparison with cardiac mr imaging. Radiology 280:49–57. https://doi.org/10.1148/radiol.2016151289

65. Si-Mohamed SA, Congi A, Ziegler A et al (2021) Early prediction of cardiac complications in acute myocarditis by means of extracellular volume quantification with the use of dual-energy computed tomography. JACC Cardiovasc Imaging 14:2041–2042. https://doi.org/10.1016/j.jcmg.2021.04.008

66. Ohta Y, Kitao S, Yunaga H et al (2019) Quantitative evaluation of non-ischemic dilated cardiomyopathy by late iodine enhancement using rapid kv switching dual-energy computed tomography: a feasibility study. J Cardiovasc Comput Tomogr 13:148–156. https://doi.org/10.1016/j.jcct.2018.10.028

67. Vignale D, Palmisano A, Gnasso C et al (2023) Extracellular volume fraction (ecv) derived from pre-operative computed tomography predicts prognosis in patients undergoing transcatheter aortic valve implantation (tavi). Eur Heart J Cardiovasc Imaging 24:887–896. https://doi.org/10.1093/ehjci/jead040

68. Ogata F, Hanatani S, Nakashima N et al (2024) Human epididymis protein 4 is a useful predictor of post-operative prognosis in patients with severe aortic stenosis. ESC Heart Fail 11:2924–2932. https://doi.org/10.1002/ehf2.14845

69. Tavoosi A, Brito JB de O, El Mais H et al (2023) Dual versus single energy cardiac ct to measure extra cellular volume in cardiac amyloidosis: correlations with cardiac mri. IJC Heart and Vasculature 44:101166. https://doi.org/10.1016/j.ijcha.2022.101166

70. Kidoh M, Oda S, Takashio S et al (2023) CT extracellular volume fraction versus myocardium-to-lumen signal ratio for cardiac amyloidosis. Radiology 306:e220542. https://doi.org/10.1148/radiol.220542

71. Wang T, Ouyang B, Tang L, Li L, Liu Y (2024) Myocardial extracellular volume derived from contrast-enhanced chest computed tomography in longitudinal evaluation of cardiac toxicity in patients treated with immune checkpoint inhibitors. Quant Imaging Med Surg 14:3107–3120. https://doi.org/10.21037/qims-23-850

72. Mirelis JG, Sánchez-González J, Zorio E et al (2017) Myocardial extracellular volume is not associated with malignant ventricular arrhythmias in high-risk hypertrophic cardiomyopathy. Revista Española de Cardiología (English Edition) 70:933–940. https://doi.org/10.1016/j.rec.2017.01.026

73. Hammer Y, Barkan YT, Abelow A et al (2021) Myocardial extracellular volume quantification by computed tomography predicts outcomes in patients with severe aortic stenosis. PLoS One 16:e0248306. https://doi.org/10.1371/journal.pone.0248306

74. Patel KP, Scully PR, Saberwal B et al (2024) Regional distribution of extracellular volume quantified by cardiac ct in aortic stenosis: insights into disease mechanisms and impact on outcomes. Circ Cardiovasc Imaging 17:e015996. https://doi.org/10.1161/CIRCIMAGING.123.015996

75. Esposito A, Palmisano A, Antunes S et al (2018) Assessment of remote myocardium heterogeneity in patients with ventricular tachycardia using texture analysis of late iodine enhancement (lie) cardiac computed tomography (cct) images. Mol Imaging Biol 20:816–825. https://doi.org/10.1007/s11307-018-1175-1

76. Morioka T, Kato S, Onoma A et al (2024) Improvement of quantification of myocardial synthetic ecv with second-generation deep learning reconstruction. J Cardiovasc Dev Dis 11:304. https://doi.org/10.3390/jcdd11100304

77. Jiang Y, Ye J, Yang Y et al (2024) Prognostic value of measurement of myocardial extracellular volume using dual-energy ct in heart failure with preserved ejection fraction. Sci Rep 14:7504. https://doi.org/10.1038/s41598-024-58271-9

78. Tamarappoo B, Han D, Tyler J et al (2020) Prognostic value of computed tomography–derived extracellular volume in tavr patients with low-flow low-gradient aortic stenosis. JACC Cardiovasc Imaging 13:2591–2601. https://doi.org/10.1016/j.jcmg.2020.07.045

79. Oda S, Emoto T, Nakaura T et al (2019) Myocardial late iodine enhancement and extracellular volume quantification with dual-layer spectral detector dual-energy cardiac ct. Radiol Cardiothorac Imaging 1:e180003. https://doi.org/10.1148/ryct.2019180003

80. Tu C, Shen H, Liu R et al (2022) Myocardial extracellular volume derived from contrast-enhanced chest computed tomography for longitudinal evaluation of cardiotoxicity in patients with breast cancer treated with anthracyclines. Insights Imaging 13:85. https://doi.org/10.1186/s13244-022-01224-5

81. Han D, Tamarappoo B, Klein E et al (2021) Computed tomography angiography-derived extracellular volume fraction predicts early recovery of left ventricular systolic function after transcatheter aortic valve replacement. Eur Heart J Cardiovasc Imaging 22:179–185. https://doi.org/10.1093/ehjci/jeaa310

82. Scully PR, Morris E, Patel KP et al (2020) DPD quantification in cardiac amyloidosis: a novel imaging biomarker. JACC Cardiovasc Imaging 13:1353–1363. https://doi.org/10.1016/j.jcmg.2020.03.020

83. Suzuki M, Toba T, Izawa Y et al (2021) Prognostic impact of myocardial extracellular volume fraction assessment using dual-energy computed tomography in patients treated with aortic valve replacement for severe aortic stenosis. J Am Heart Assoc 10:e020655. https://doi.org/10.1161/JAHA.120.020655

84. Qi RX, Shao J, Jiang JS et al (2021) Myocardial extracellular volume fraction quantitation using cardiac dual-energy ct with late iodine enhancement in patients with heart failure without coronary artery disease: a single-center prospective study. Eur J Radiol 140:109743. https://doi.org/10.1016/j.ejrad.2021.109743

85. Monti CB, Zanardo M, Capra D et al (2023) Myocardial extracellular volume assessment at ct in hospitalized covid-19 patients with regards to pulmonary embolism. Eur J Radiol 163:110809. https://doi.org/10.1016/j.ejrad.2023.110809

86. Ohta Y, Kishimoto J, Kitao S et al (2020) Investigation of myocardial extracellular volume fraction in heart failure patients using iodine map with rapid-kv switching dual-energy ct: segmental comparison with mri t1 mapping. J Cardiovasc Comput Tomogr 14:349–355. https://doi.org/10.1016/j.jcct.2019.12.032

87. Wang R, Liu X, Schoepf UJ et al (2018) Extracellular volume quantitation using dual-energy ct in patients with heart failure: comparison with 3t cardiac mr. Int J Cardiol 268:236–240. https://doi.org/10.1016/j.ijcard.2018.05.027

88. Kim NY, Im DJ, Youn JC et al (2022) Synthetic extracellular volume fraction derived using virtual unenhanced attenuation of blood on contrast-enhanced cardiac dual-energy ct in nonischemic cardiomyopathy. American Journal of Roentgenology 218:454–461. https://doi.org/10.2214/AJR.21.26654

89. Esposito F, Mezzanotte V, Tesei C et al (2024) CT images in follicular lymphoma: changes after treatment are predictive of cardiac toxicity in patients treated with anthracycline-based or r-b regimens. Cancers (Basel) 16:563. https://doi.org/10.3390/cancers16030563

90. Treibel TA, Bandula S, Fontana M et al (2015) Extracellular volume quantification by dynamic equilibrium cardiac computed tomography in cardiac amyloidosis. J Cardiovasc Comput Tomogr 9:585–592. https://doi.org/10.1016/j.jcct.2015.07.001

91. Mergen V, Ehrbar N, Moser LJ et al (2024) Synthetic hematocrit from virtual non-contrast images for myocardial extracellular volume evaluation with photon-counting detector ct. Eur Radiol 34:7845–7855. https://doi.org/10.1007/s00330-024-10865-7

92. Nowak J, Sikora M, Drabik M et al (2024) Computed tomography-derived myocardial extracellular volume fraction — a redundant by-product or a novel promising marker? Folia Med Cracov 64:129–135. https://doi.org/10.24425/fmc.2024.152173

93. Nakato K, Oda S, Yamaguchi S et al (2025) Cardiac ct-derived quantification of myocardial extracellular volume using deep learning-based reconstruction: a feasibility study. J Cardiovasc Comput Tomogr 19:83–84. https://doi.org/10.1016/j.jcct.2024.07.008

94. Egashira K, Sueta D, Kidoh M et al (2022) Cardiac computed tomography-derived myocardial tissue characterization after anthracycline treatment. ESC Heart Fail 9:1792–1800. https://doi.org/10.1002/ehf2.13867

95. Kumar V, Harfi TT, He X et al (2019) Estimation of myocardial fibrosis in humans with dual energy ct. J Cardiovasc Comput Tomogr 13:315–318. https://doi.org/10.1016/j.jcct.2018.12.004

96. Takahashi M, Takaoka H, Yashima S et al (2024) Extracellular volume fraction by computed tomography predicts prognosis after transcatheter aortic valve replacement. Circulation Journal 88:492–500. https://doi.org/10.1253/circj.CJ-23-0288

97. Nishigake D, Yamasaki Y, Hida T et al (2024) Influence of cardiac cycle on myocardial extracellular volume fraction measurements with dual-layer computed tomography. Quant Imaging Med Surg 14:4714–4722. https://doi.org/10.21037/qims-23-1647

98. Yamasaki H, Kondo H, Shiroo T et al (2024) Efficacy of computed tomography-based evaluation of myocardial extracellular volume combined with red flags for early screening of concealed cardiac amyloidosis in patients with atrial fibrillation. Circulation Journal 88:1167–1175. https://doi.org/10.1253/circj.CJ-23-0948

99. Liang J, Li H, Xie J et al (2022) Iodine-based extracellular volume for evaluating myocardial status in patients undergoing percutaneous coronary intervention for acute myocardial infarction by using dual-layer

spectral detector computed tomography: a comparison study with magnetic resonance. Quant Imaging Med Surg 12:4502–4511. https://doi.org/10.21037/qims-21-1103

100. Oda S, Kidoh M, Takashio S et al (2020) Quantification of myocardial extracellular volume with planning computed tomography for transcatheter aortic valve replacement to identify occult cardiac amyloidosis in patients with severe aortic stenosis. Circ Cardiovasc Imaging 13:e010358. https://doi.org/10.1161/CIRCIMAGING.119.010358

101. Abadia AF, van Assen M, Martin SS et al (2020) Myocardial extracellular volume fraction to differentiate healthy from cardiomyopathic myocardium using dual-source dual-energy ct. J Cardiovasc Comput Tomogr 14:162–167. https://doi.org/10.1016/j.jcct.2019.09.008

102. Gnasso C, Pinos D, Schoepf UJ et al (2024) Impact of reconstruction parameters on the accuracy of myocardial extracellular volume quantification on a first-generation, photon-counting detector ct. Eur Radiol Exp 8:70. https://doi.org/10.1186/s41747-024-00469-7

103. Kidoh M, Oda S, Takashio S et al (2020) Assessment of diffuse ventricular fibrosis in atrial fibrillation using cardiac ct-derived myocardial extracellular volume fraction. JACC Clin. Electrophysiol. 6:1573–1575

104. Sueta D, Kidoh M, Oda S et al (2021) Usefulness of cardiac computed tomography in the diagnosis of anti-cancer therapy-related cardiac dysfunction - consistency with magnetic resonance imaging. Circulation Journal 85:393–396. https://doi.org/10.1253/CIRCJ.CJ-20-1288

105. Kurita Y, Kitagawa K, Kurobe Y et al (2016) Estimation of myocardial extracellular volume fraction with cardiac ct in subjects without clinical coronary artery disease: a feasibility study. J Cardiovasc Comput Tomogr 10:237–241. https://doi.org/10.1016/j.jcct.2016.02.001

106. Takafuji M, Kitagawa K, Nakamura S et al (2020) Feasibility of extracellular volume fraction calculation using myocardial ct delayed enhancement with low contrast media administration. J Cardiovasc Comput Tomogr 14:524–528. https://doi.org/10.1016/j.jcct.2020.01.013

107. Goto H, Takaoka H, Ota J et al (2024) Computed tomography evaluation of extracellular volume for predicting prognosis in patients with severe renal dysfunction on dialysis. J Clin Med 13:7561. https://doi.org/10.3390/jcm13247561

108. Rosenfeld R, Riondino S, Cerocchi M et al (2024) Extracellular volume measured by whole body ct scans predicts chronic cardiotoxicity in breast cancer patients treated with neoadjuvant therapies based on anthracyclines: a retrospective study. Breast 76:103755. https://doi.org/10.1016/j.breast.2024.103755

109. Oguni T, Takashio S, Kuyama N et al (2024) Clinical characteristics of patients with high extracellular volume fraction evaluated by cardiac computed tomography for coronary artery evaluation. European Heart Journal Open 4:oeae036. https://doi.org/10.1093/ehjopen/oeae036

110. Chiocchi M, Cerocchi M, Di Tosto F et al (2023) Quantification of extracellular volume in ct in neoadjuvant chemotherapy in breast cancer: new frontiers in assessing the cardiotoxicity of anthracyclines and trastuzumab. J Pers Med 13:199. https://doi.org/10.3390/jpm13020199

111. Dubourg B, Dacher JN, Durand E et al (2021) Single-source dual energy ct to assess myocardial extracellular volume fraction in aortic stenosis before transcatheter aortic valve implantation (tavi). Diagn Interv Imaging 102:561–570. https://doi.org/10.1016/j.diii.2021.03.003

112. Li N, Zhang X, Gu J et al (2024) Quantitating myocardial fibrosis using extracellular extravascular volume determined from computed tomography myocardial perfusion imaging. BMC Med Imaging 24:40. https://doi.org/10.1186/s12880-024-01226-3

113. Emoto T, Kidoh M, Oda S et al (2020) Myocardial extracellular volume quantification in cardiac ct: comparison of the effects of two different iterative reconstruction algorithms with mri as a reference standard. Eur Radiol 30:691–701. https://doi.org/10.1007/s00330-019-06418-y

Table S1 – Data extraction

ARTICLES DATA							TECHNICAL DATA												
N.	AUTHOR	YEAR	COUNTRY	TITLE	JOURNAL	STUDY DESIGN	CLINICAL SETTING	SAMPLE SIZE	CT TYPE	ACQUISITION APPROACH	DELAY TIMING (MIN)	CONTRAST PROTOCOL	HEMATOCRIT	ROI METHOD	COMPARATOR	ECG GATING	ECV CUT-OFF	ECV CUT-OFF VALUE	ECV CUT-OFF USE
1	Kengo Nakato	2025	Japan	Cardiac CT-Derived Quantification Of Myocardial Extracellular Volume Using Deep Learning-Based Reconstruction: A Feasibility Study	JOURNAL OF CARDIOVASCULAR COMPUTED TOMOGRAPHY	RETROSPECTIVE	VARIOUS CARDIAC DISEASE	39	SECT	PRE-CONTRAST + DELAYED	7'	550 mgI/kg	NOT REPORTED	LEFT VENTRICLE	CMR	NOT REPORTED	NO	NO + NOT APPLICABLE	NOT APPLICABLE
2	Masafumi Kidoh	2020	Japan	Assessment Of Diffuse Ventricular Fibrosis In Atrial Fibrillation Using Cardiac CT-Derived Myocardial Extracellular Volume Fraction	JOURNAL OF THE AMERICAN COLLEGE OF CARDIOLOGY	RETROSPECTIVE	VARIOUS CARDIAC DISEASE	105	SECT	PRE-CONTRAST + DELAYED	NOT REPORTED	NOT REPORTED	NOT REPORTED	NOT REPORTED	NONE/FEASIBILITY ONLY	NOT REPORTED	NO	NO + NOT APPLICABLE	NOT APPLICABLE
3	Salim A Si-Mohamed	2021	France	Early Prediction Of Cardiac Complications In Acute Myocarditis By Means Of Extracellular Volume Quantification With The Use Of Dual-Energy Computed Tomography	JOURNAL OF THE AMERICAN COLLEGE OF CARDIOLOGY	RETROSPECTIVE	MYOCARDITIS	61	DECT	IODINE-MAP BASED	10'	480 mgI/kg	MEASURED	NOT REPORTED	NONE/FEASIBILITY ONLY	NOT REPORTED	YES	39.50%	MAJOR ADVERSE CARDIOVASCULAR EVENT
4	Seitaro Oda	2020	Japan	Quantification Of Myocardial Extracellular Volume With Planning Computed Tomography For Transcatheter Aortic Valve Replacement To Identify Occult Cardiac Amyloidosis In Patients With Severe Aortic Stenosis	CIRCULATION CARDIOVASCULAR IMAGING	RETROSPECTIVE	AORTIC STENOSIS AND AMYLOIDOSIS	192	SECT	PRE-CONTRAST + DELAYED	7'	550 mgI/kg	NOT REPORTED	NOT REPORTED	SCINTIGRAPHY	NOT REPORTED	YES	33.70%	POST-OPERATIVE PROGNOSIS
5	Victor Mergen	2022	Switzerland	Extracellular Volume Quantification With Cardiac Late Enhancement Scanning Using Dual-Source Photon-Counting Detector CT	INVESTIGATIVE RADIOLOGY	PROSPECTIVE	AORTIC STENOSIS	30	PCCT	IODINE-MAP BASED	5'	100 ml	MEASURED	NOT REPORTED	NONE/FEASIBILITY ONLY	GATED	NO	NO + NOT APPLICABLE	NOT APPLICABLE
6	Francisco Gama	2022	UK	Extracellular Volume Fraction By Computed Tomography Predicts Long-Term Prognosis Among Patients With Cardiac Amyloidosis	JOURNAL OF THE AMERICAN COLLEGE OF CARDIOLOGY	PROSPECTIVE	AMYLOIDOSIS	72	SECT	PRE-CONTRAST + DELAYED	5'	300 mgI/kg	MEASURED	NOT REPORTED	NONE/FEASIBILITY ONLY	GATED	NO	NO + NOT APPLICABLE	NOT APPLICABLE
7	Masafumi Kidoh	2023	Japan	CT Extracellular Volume Fraction Versus Myocardium-To-Lumen Signal Ratio For Cardiac Amyloidosis	RADIOLOGY	RETROSPECTIVE	AMYLOIDOSIS	552	SECT	PRE-CONTRAST + DELAYED	7'	550 mgI/kg	MEASURED	SEPTAL/LEFT VENTRICLE	HISTOLOGY	NOT REPORTED	YES	37.00%	DIAGNOSIS

**8**	Koichi Egashira	2021	Japan	Cardiac Computed Tomography-Derived Extracellular Volume Fraction In Late Anthracycline-Induced Cardiotoxicity	INTERNATIONAL JOURNAL OF CARDIOLOGY	PROSPECTIVE	ONCOLOGY	44	SECT	NOT REPORTED	NOT REPORTED	NOT REPORTED	NOT REPORTED	NOT REPORTED	NONE/FEASIBILITY ONLY	NOT REPORTED	NO	NO + NOT APPLICABLE	NOT APPLICABLE
**9**	Koichi Egashira	2022	Japan	Cardiac Computed Tomography-Derived Myocardial Tissue Characterization After Anthracycline Treatment	ESC HEART FAILURE	PROSPECTIVE	ONCOLOGY	15	SECT	PRE-CONTRAST + DELAYED	7'	550 mgI/kg	NOT REPORTED	NOT REPORTED	NONE/FEASIBILITY ONLY	GATED	NO	NO + NOT APPLICABLE	NOT APPLICABLE
**10**	Manami Takahashi	2024	Japan	Extracellular Volume Fraction By Computed Tomography Predicts Prognosis After Transcatheter Aortic Valve Replacement	CIRCULATION JOURNAL	RETROSPECTIVE	AORTIC STENOSIS	127	SECT	PRE-CONTRAST + DELAYED	6'	350-370 mgI/kg	MEASURED	NOT REPORTED	NONE/FEASIBILITY ONLY	GATED	YES	32.60%	POST-OPERATIVE PROGNOSIS
**11**	Caterina Beatrice Monti	2023	Italy	Myocardial Extracellular Volume Assessment At CT In Hospitalized Covid-19 Patients With Regards To Pulmonary Embolism	EUROPEAN JOURNAL OF RADIOLOGY	RETROSPECTIVE	VARIOUS	94	SECT	PRE-CONTRAST + DELAYED	1'-2'	320-370 mgI/kg	MEASURED	SEPTAL/LEFT VENTRICLE	NONE/FEASIBILITY ONLY	NON-GATED	NO	NO + NOT APPLICABLE	NOT APPLICABLE
**12**	Gilberto J Aquino	2023	USA	Myocardial Characterization With Extracellular Volume Mapping With A First-Generation Photon-Counting Detector CT With MRI Reference	RADIOLOGY	PROSPECTIVE	VARIOUS CARDIAC DISEASE	29	PCCT	PRE-CONTRAST + DELAYED	5'	100 ml	SYNTHETIC	NOT REPORTED	CMR	GATED	NO	NO + NOT APPLICABLE	NOT APPLICABLE
**13**	Jesús G Mirelis	2017	Spain	Myocardial Extracellular Volume Is Not Associated With Malignant Ventricular Arrhythmias In High-Risk Hypertrophic Cardiomyopathy	REVISTA ESPANOLA DE RADIOLOGIA	RETROSPECTIVE	CARDIOMYOPATHY	78	SECT	PRE-CONTRAST + DELAYED	25'	<200 ml	MEASURED	SEPTAL/LEFT VENTRICLE	CMR	NOT REPORTED	NO	NO + NOT APPLICABLE	NOT APPLICABLE
**14**	Jakub Nowak	2024	Poland	Computed Tomography-Derived Myocardial Extracellular Volume Fraction — A Redundant By-Product Or A Novel Promising Marker?	FOLIA MEDICA CRACOVIENSA	RETROSPECTIVE	HEART FAILURE	103	SECT	PRE-CONTRAST + DELAYED	NOT REPORTED	NOT REPORTED	MEASURED	SEPTAL/LEFT VENTRICLE	NONE/FEASIBILITY ONLY	NOT REPORTED	NO	NO + NOT APPLICABLE	NOT APPLICABLE
**15**	Satomi Yashima	2023	Japan	Evaluation Of Extracellular Volume By Computed Tomography Is Useful For Prediction Of Prognosis In Dilated Cardiomyopathy	HEART AND VESSELS	RETROSPECTIVE	CARDIOMYOPATHY	70	SECT	PRE-CONTRAST + DELAYED	6'	45-100 ml	MEASURED	LEFT VENTRICLE	NONE/FEASIBILITY ONLY	GATED	YES	NO + NOT APPLICABLE	MAJOR ADVERSE CARDIOVASCULAR EVENT
**16**	Victor Mergen	2024	Switzerland	Synthetic Hematocrit From Virtual Non-Contrast Images For Myocardial Extracellular Volume Evaluation With Photon-Counting Detector CT	EUROPEAN JOURNAL OF RADIOLOGY	RETROSPECTIVE	AORTIC STENOSIS	125	PCCT	PRE-CONTRAST + DELAYED	5'	60-80 ml	SYNTHETIC	LEFT VENTRICLE/ AORTA	NONE/FEASIBILITY ONLY	GATED	NO	NO + NOT APPLICABLE	NOT APPLICABLE
**17**	Yoav Hammer	2021	Israel	Myocardial Extracellular Volume Quantification By Computed Tomography Predicts Outcomes In Patients With Severe Aortic Stenosis	PLOS ONE	PROSPECTIVE	AORTIC STENOSIS	94	SECT	PRE-CONTRAST + DELAYED	7'	50-60 ml	MEASURED	SEPTAL/LATERAL WALL/AORTA	NONE/FEASIBILITY ONLY	GATED	YES	40.00%	HOSPITALIZATION AND ALL-CAUSE MORTALITY

**18**	Hirochika Yamasaki	2024	Japan	Efficacy Of Computed Tomography-Based Evaluation Of Myocardial Extracellular Volume Combined With Red Flags For Early Screening Of Concealed Cardiac Amyloidosis In Patients With Atrial Fibrillation	CIRCULATION JOURNAL	PROSPECTIVE	AMYLOIDOSIS	342	SECT	PRE-CONTRAST + DELAYED	7'	600 mgI/kg	NOT REPORTED	VENTRICULAR SEPTUM	SCINTIGRAPHY	NOT REPORTED	YES	35.00%	DIAGNOSIS
**19**	Vidhya Kumar	2019	USA	Estimation Of Myocardial Fibrosis In Humans With Dual Energy CT	JOURNAL OF CARDIOVASCULAR COMPUTED TOMOGRAPHY	PROSPECTIVE	VARIOUS CARDIAC DISEASE	28	DECT	ATTENUATION-BASED	NOT REPORTED	NOT REPORTED	NOT REPORTED	MID-INFEROSEPTAL SEGMENT/LEFT VENTRICLE	CMR	GATED	YES	29.00%	DIAGNOSIS
**20**	Ying Jiang	2024	China	Prognostic Value Of Measurement Of Myocardial Extracellular Volume Using Dual-Energy CT In Heart Failure With Preserved Ejection Fraction	SCIENTIFIC REPORTS	RETROSPECTIVE	HEART FAILURE	125	DECT	PRE-CONTRAST + DELAYED	7'	50 ml	MEASURED	LEFT VENTRICLE	NONE/FEASIBILITY ONLY	GATED	NO	NO + NOT APPLICABLE	NOT APPLICABLE
**21**	Na Li	2024	China	Quantitating Myocardial Fibrosis Using Extracellular Extravascular Volume Determined From Computed Tomography Myocardial Perfusion Imaging	BMC MEDICAL IMAGING	PROSPECTIVE	VARIOUS CARDIAC DISEASE	17	SECT	PRE-CONTRAST + DELAYED	5'	400 mgI/ml x 0.7 ml/kg	NOT REPORTED	NOT REPORTED	CMR	NOT REPORTED	YES	41.00%	DIAGNOSIS
**22**	Davide Vignale	2023	Italy	Extracellular Volume Fraction (ECV) Derived From Pre-Operative Computed Tomography Predicts Prognosis In Patients Undergoing Transcatheter Aortic Valve Implantation (TAVI)	EUROPEAN HEART JOURNAL CARDIOVASCULAR IMAGING	PROSPECTIVE	AORTIC STENOSIS	159	SECT	PRE-CONTRAST + DELAYED	5'	85-110 ml	NOT REPORTED	SEPTUM/LEFT VENTRICLE	NONE/FEASIBILITY ONLY	GATED	YES	31.30%	HEART FAILURE HOSPITALIZATION AND DEATH
**23**	Kush P Patel	2024	UK	Regional Distribution Of Extracellular Volume Quantified By Cardiac CT In Aortic Stenosis: Insights Into Disease Mechanisms And Impact On Outcomes	CIRCULATION CARDIOVASCULAR IMAGING	PROSPECTIVE	AORTIC STENOSIS	199	SECT	PRE-CONTRAST + DELAYED	3'	NOT REPORTED	NOT REPORTED	GLOBAL AND REGIONAL	NONE/FEASIBILITY ONLY	GATED	NO	NO + NOT APPLICABLE	NOT APPLICABLE
**24**	Thomas A Treibel	2015	UK	Extracellular Volume Quantification By Dynamic Equilibrium Cardiac Computed Tomography In Cardiac Amyloidosis	JOURNAL OF CARDIOVASCULAR COMPUTED TOMOGRAPHY	PROSPECTIVE	AMYLOIDOSIS	26	SECT	PRE-CONTRAST + DELAYED	5' and 15'	652 mgI/kg	MEASURED	SEPTUM/LEFT VENTRICLE	NONE/FEASIBILITY ONLY	GATED	NO	NO + NOT APPLICABLE	NOT APPLICABLE
**25**	Balaji Tamarappoo	2020	USA	Prognostic Value Of Computed Tomography–Derived Extracellular Volume In Tavr Patients With Low-Flow Low-Gradient Aortic Stenosis	JOURNAL OF THE AMERICAN COLLEGE OF CARDIOLOGY	RETROSPECTIVE	AORTIC STENOSIS	150	SECT	PRE-CONTRAST + DELAYED	5'	100 ml	NOT REPORTED	SEPTUM AND LATERAL WALL IN THE BASAL AND MID-LV/LEFT VENTRICLE	NONE/FEASIBILITY ONLY	GATED	YES	33.00%	HOSPITALIZATION AND ALL-CAUSE MORTALITY
**26**	Masaki Ishiyama	2023	Japan	The Cardiac Computed Tomography-Derived Extracellular Volume Fraction Predicts Patient Outcomes And	JOURNAL OF CARDIOLOGY	RETROSPECTIVE	AORTIC STENOSIS	71	SECT	PRE-CONTRAST + DELAYED	5'	26 mgI/kg/s	NOT REPORTED	LEFT VENTRICLE	NONE/FEASIBILITY ONLY	NOT REPORTED	YES	35.00%	HOSPITALIZATION AND ALL-CAUSE MORTALITY

				Left Ventricular Mass Reductions After Transcatheter Aortic Valve Implantation For Aortic Stenosis															
**27**	Hideki Koike	2024	USA	Comprehensive Myocardial Assessment By Computed Tomography	JOURNAL OF THE AMERICAN COLLEGE OF CARDIOLOGY	RETROSPECTIVE	AORTIC STENOSIS	300	SECT	PRE-CONTRAST + DELAYED	3'	100 ml	MEASURED	SEPTAL/LEFT VENTRICLE	NONE/FEASIBILITY ONLY	GATED	YES	29.00%	HOSPITALIZATION AND ALL-CAUSE MORTALITY
**28**	Salim Aymeric Si-Mohamed	2021	France	Diagnostic Performance Of Extracellular Volume Quantified By Dual-Layer Dual-Energy CT For Detection Of Acute Myocarditis	JOURNAL OF CLINICAL MEDICINE	RETROSPECTIVE	MYOCARDITIS	78	DECT	DELAYED ONLY	10'	400 mgI/ml x 1.2 ml/kg	MEASURED	LEFT VENTRICLE	NONE/FEASIBILITY ONLY	GATED	YES	31.60%	DIAGNOSIS
**29**	Donghee Han	2021	USA	Computed Tomography Angiography-Derived Extracellular Volume Fraction Predicts Early Recovery Of Left Ventricular Systolic Function After Transcatheter Aortic Valve Replacement	EUROPEAN HEART JOURNAL CARDIOVASCULAR IMAGING	RETROSPECTIVE	AORTIC STENOSIS	109	SECT	PRE-CONTRAST + DELAYED	5'	100 ml	NOT REPORTED	SEPTUM AND LATERAL WALL IN THE BASAL AND MID LV/LEFT VENTRICLE	NONE/FEASIBILITY ONLY	GATED	YES	30.00%	LEFT VENTRICULAR EJECTION FRACTION RECOVERY
**30**	Jean-François Deux	2021	France	Diagnostic Value Of Extracellular Volume Quantification And Myocardial Perfusion Analysis At CT In Cardiac Amyloidosis	RADIOLOGY	PROSPECTIVE	AMYLOIDOSIS	160	SECT	PRE-CONTRAST + DELAYED	5'	320 mgI/ml x 1.5 ml/kg	MEASURED	NOT REPORTED	NONE/FEASIBILITY ONLY	NOT REPORTED	YES	55.00%	SURVIVAL
**31**	Daisuke Sueta	2021	Japan	Usefulness Of Cardiac Computed Tomography In The Diagnosis Of Anti-Cancer Therapy-Related Cardiac Dysfunction	CIRCULATION JOURNAL	PROSPECTIVE	ONCOLOGY	7	SECT	PRE-CONTRAST + DELAYED	NOT REPORTED	NOT REPORTED	NOT REPORTED	NOT REPORTED	CMR	NOT REPORTED	NO	NO + NOT APPLICABLE	NOT APPLICABLE
**32**	Masafumi Takafuji	2020	Japan	Feasibility Of Extracellular Volume Fraction Calculation Using Myocardial CT Delayed Enhancement With Low Contrast Media Administration	JOURNAL OF CARDIOVASCULAR COMPUTED TOMOGRAPHY	RETROSPECTIVE	VARIOUS CARDIAC DISEASE	20	SECT	PRE-CONTRAST + DELAYED	NOT REPORTED	370 mgI/ml x 0.84 ml/kg (+40 ml)	MEASURED	LEFT VENTRICLE	NONE/FEASIBILITY ONLY	GATED	NO	NO + NOT APPLICABLE	NOT APPLICABLE
**33**	Chunrong Tu	2022	China	Myocardial Extracellular Volume Derived From Contrast-Enhanced Chest Computed Tomography For Longitudinal Evaluation Of Cardiotoxicity In Patients With Breast Cancer Treated With Anthracyclines	INSIGHTS INTO IMAGING	RETROSPECTIVE	ONCOLOGY	1151	SECT	PRE-CONTRAST + DELAYED	80''	320 mgI/ml x 1.5 ml/kg	MEASURED	MIDDLE OF INTERVENTRICULAR SEPTUM/LEFT VENTRICLE	NONE/FEASIBILITY ONLY	NON-GATED	NO	NO + NOT APPLICABLE	NOT APPLICABLE
**34**	Yoshito Kadoya	2025	Canada	Reproducibility Of Myocardial Extracellular Volume Quantification Using Dual-Energy Computed Tomography In Patients With Cardiac Amyloidosis	JOURNAL OF CARDIOVASCULAR COMPUTED TOMOGRAPHY	PROSPECTIVE	AMYLOIDOSIS	24	DECT	PRE-CONTRAST + DELAYED	7' and 8'	350 mgI/ml x 1 ml/kg	MEASURED	NOT REPORTED	NONE/FEASIBILITY ONLY	GATED	NO	NO + NOT APPLICABLE	NOT APPLICABLE
**35**	Rui Wang	2018	China, USA, Netherlands, Australia	Extracellular Volume Quantification Using Dual-Energy CT In Patients With Heart Failure: Comparison With 3T Cardiac MR	INTERNATIONAL JOURNAL OF CARDIOLOGY	PROSPECTIVE	HEART FAILURE	35	DECT	PRE-CONTRAST + DELAYED	7'	60-90 ml	MEASURED	LEFT VENTRICLE	CMR	GATED	NO	NO + NOT APPLICABLE	NOT APPLICABLE

**36**	R Rosenfeld	2024	Italy	Extracellular Volume Measured By Whole Bofy Ct Scans Predicts Chronic Cardiotoxicity In Breast Cancer Patients Treated With Neoadjuvant Therapies Based On Anthracyclines: A Retrospective Study	BREAST	RETROSPECTIVE	ONCOLOGY	82	SECT	PRE-CONTRAST + DELAYED	5'	100-120 ml	MEASURED	MIDDLE SEPTUM/LEFT VENTRICLE	NONE/FEASIBILITY ONLY	NON-GATED	YES	35.00%	12-FOLD INCREASED RISK OF DEVELOPING CTRCD
**37**	Anahita Tavoosi	2022	Canada, Brazil	Dual Versus Single Energy Cardiac CT To Measure Extracellular Volume In Cardiac Amyloidosis: Correlations With Cardiac MRI	INTERNATIONAL JOURNAL OF CARDIOLOGY	PROSPECTIVE	AMYLOIDOSIS	25	DECT	PRE-CONTRAST + DELAYED	5'	NOT REPORTED	MEASURED	BASAL TO MID LV INFERIOR SEPTUM/LEFT VENTRICLE	CMR	GATED	NO	NO + NOT APPLICABLE	NOT APPLICABLE
**38**	Andres F Abadia	2020	USA, The Netherlands, Germany	Myocardial Extracellular Volume Fraction To Differentiate Healthy From Cardiomyopathic Myocardium Using Dual-Source Dual-Energy Ct	JOURNAL OF CARDIOVASCULAR COMPUTED TOMOGRAPHY	RETROSPECTIVE	CARDIOMYOPATHY	70	DECT	PRE-CONTRAST + DELAYED	7'	70 ml	MEASURED	SEPTAL/LEFT VENTRICLE	NONE/FEASIBILITY ONLY	GATED	YES	29.50%	DIAGNOSIS
**39**	Marcello Chiocchi	2017	Italy	Quantification Of Extracellular Volume In CT In Neoadjuvant Chemotherapy In Breast Cancer: New Frontiers In Assessing The Cardiotoxicity Of Anthracyclines And Trastuzumab	JOURNAL OF PERSONALIZED MEDICINE	RETROSPECTIVE	ONCOLOGY	102	SECT	PRE-CONTRAST + DELAYED	5'	100-120 ml	MEASURED	MIDDLE SEPTUM/LEFT VENTRICLE	NONE/FEASIBILITY ONLY	NON-GATED	NO	NO + NOT APPLICABLE	NOT APPLICABLE
**40**	Benjamin Dubourg	2021	France	Single-Source Dual Energy Ct To Assess Myocardial Extracellular Volume Fraction In Aortic Stenosis Before Transcatheter Aortic Valve Implantation (TAVI)	DIAGNOSTIC AND INTERVENTIONAL IMAGING	RETROSPECTIVE	AORTIC STENOSIS	21	DECT	PRE-CONTRAST + DELAYED	7'	65 ml	MEASURED	LEFT VENTRICLE/ RIGHT ATRIUM	CMR	GATED	NO	NO + NOT APPLICABLE	NOT APPLICABLE
**41**	Hiroki Goto	2024	Japan	Computed Tomography Evaluation Of Extracellular Volume For Predicting Prognosis In Patients With Severe Renal Dysfunction On Dialysis	JOURNAL OF CLINICAL MEDICINE	RETROSPECTIVE	VARIOUS	57	SECT	PRE-CONTRAST + DELAYED	6'	50-100 ml	MEASURED	LEFT VENTRICLE	NONE/FEASIBILITY ONLY	GATED	YES	37.26%	MAJOR ADVERSE CARDIOVASCULAR EVENT
**42**	Takafumi Emoto	2020	Japan	Myocardial Extracellular Volume Quantification In Cardiac CT: Comparison Of The Effects Of Two Different Iterative Reconstruction Algorithms With MRI As A Reference Standard	EUROPEAN RADIOLOGY	RETROSPECTIVE	VARIOUS CARDIAC DISEASE	34	SECT	PRE-CONTRAST + DELAYED	7'	500 mgI/kg	MEASURED	GLOBAL MID-VENTRICULAR WALL	CMR	GATED	NO	NO + NOT APPLICABLE	NOT APPLICABLE
**43**	Daisuke Nishigake	2024	Japan	Influence Of Cardiac Cycle On Myocardial Extracellular Volume Fraction Measurements With Dual-Layer Computed Tomography	QUANTITATIVE IMAGING IN MEDICINE AND SURGERY	RETROSPECTIVE	VARIOUS CARDIAC DISEASE	55	SECT	PRE-CONTRAST + DELAYED	10'	80-100 ml	NOT REPORTED	RIGHT AND LEFT VENTRICLE	NONE/FEASIBILITY ONLY	GATED	NO	NO + NOT APPLICABLE	NOT APPLICABLE

**44**	Paul R Scully	2020	UK	Identifying Cardiac Amyloid In Aortic Stenosis	JOURNAL OF THE AMERICAN COLLEGE OF CARDIOLOGY	RETROSPECTIVE	AMYLOIDOSIS	109	DECT	PRE-CONTRAST + DELAYED	3' and 5'	90 ml	MEASURED	NOT REPORTED	SCINTIGRAPHY	GATED	YES	31,00%	POSSIBLE DIAGNOSIS
**45**	Akimasa Yamada	2020	Japan	Quantification Of Extracellular Volume Fraction By Cardiac Computed Tomography For Noninvasive Assessment Of Myocardial Fibrosis In Hemofialysis Patients	SCIENTIFIC REPORTS	RETROSPECTIVE	VARIOUS	40	SECT	PRE-CONTRAST + DELAYED	7'	40 ml	MEASURED	LEFT VENTRICLE	NONE/FEASIBILITY ONLY	GATED	NO	NO + NOT APPLICABLE	NOT APPLICABLE
**46**	Na Young Kim	2022	South Korea	Synthetic Extracellular Volume Fraction Derived Using Virtual Unenhanced Attenuation Of Bloof On Contrast-Enhanced Cardiac Dual-Energy CT In Nonischemic Cardiomyopathy	AMERICAN JOURNAL OF ROENTEROLOGY	RETROSPECTIVE	CARDIOMYOPATHY	51	DECT	DELAYED ONLY	12'	370 mgI/ml x 1 ml/kg	SYNTHETIC	SEPTUM OF THE MID LV/LEFT VENTRICLE	CMR	GATED	NO	NO + NOT APPLICABLE	NOT APPLICABLE
**47**	Takafumi Emoto	2021	Japan	Myocardial Extracellular Volume Quantification Using Cardiac Computed Tomography: A Comparison Of The Dual-Energy iodine Method And The Standard Subtraction Method	ACADEMIC RADIOLOGY	RETROSPECTIVE	VARIOUS CARDIAC DISEASE	21	DECT	PRE-CONTRAST + DELAYED	7'	550 mgI/kg	NOT REPORTED	LEFT VENTRICLE	CMR	GATED	NO	NO + NOT APPLICABLE	NOT APPLICABLE
**48**	Jing Liang	2022	China	Iodine-Based Extracellular Volume For Evaluating Myocardial Status In Patients Undergoing Percutaneous Coronary Intervention For Acute Myocardial Infarction By Using Dual-Layer Spectral Detector Computed Tomography: A Comparison Study With Magnetic Resonance	QUANTITATIVE IMAGING IN MEDICINE AND SURGERY	PROSPECTIVE	VARIOUS CARDIAC DISEASE	21	SECT	PRE-CONTRAST + DELAYED	8'	350 mgI/ml x 0.9-1.0 mg/kg	MEASURED	LEFT VENTRICLE	CMR	GATED	NO	NO + NOT APPLICABLE	NOT APPLICABLE
**49**	Yoshie Kurita	2016	Japan	Estimation Of Myocardial Extracellular Volume Fraction With Cardiac CT In Subjects Without Clinical Coronary Artery Disease: A Feasibility Study	JOURNAL OF CARDIOVASCULAR COMPUTED TOMOGRAPHY	RETROSPECTIVE	VARIOUS CARDIAC DISEASE	38	SECT	PRE-CONTRAST + DELAYED	7'	60 ml	MEASURED	LEFT VENTRICLE	NONE/FEASIBILITY ONLY	GATED	NO	NO + NOT APPLICABLE	NOT APPLICABLE
**50**	Rong-Xing Qi	2021	China	Measurement Of Myocardial Extracellular Volume Fraction In Patients With Heart Failure With Preserved Ejection Fraction Using Dual-Energy Computed Tomography	EUROPEAN JOURNAL OF RADIOLOGY	PROSPECTIVE	HEART FAILURE	192	DECT	DELAYED ONLY	7'	50 ml	NOT REPORTED	LEFT VENTRICLE	NONE/FEASIBILITY ONLY	GATED	NO	NO + NOT APPLICABLE	NOT APPLICABLE
**51**	Peijun Liu	2022	China, USA	Quantitative Analysis Of Late iodine Enhancement Using Dual-Layer Spectral Detector Computed Tomography: Comparison With	QUANTITATIVE IMAGING IN MEDICINE AND SURGERY	PROSPECTIVE	VARIOUS CARDIAC DISEASE	56	DECT	DELAYED ONLY	7'	370 mgI/ml x 0.9 ml/kg	MEASURED	LEFT VENTRICLE	NONE/FEASIBILITY ONLY	GATED	YES	31.00%	ISCHEMIC MYOCARDIAL

				Magnetic Resonance Imaging															
**52**	Seitaro Oda	2019	Japan	Myocardial Late iodine Enhancement And Extracellular Volume Quantification With Dual- Layer Spectral Detector Dual-Energy Cardiac CT	RADIOLOGY CARDIOTHORACIC IMAGING	RETROSPECTIVE	VARIOUS CARDIAC DISEASE	40	DECT	DELAYED ONLY	7'	550 mgI/kg	NOT REPORTED	LEFT VENTRICLE	CMR	GATED	NO	NO + NOT APPLICABLE	NOT APPLICABLE
**53**	Ting Wang	2024	China	Myocardial Extracellular Volume Derived From Contrast-Enhanced Chest Computed Tomography In Longitudinal Evaluation Of Cardiac Toxicity In Patients Treated With Immune Checkpoint Inhibitors	QUANTITATIVE IMAGING IN MEDICINE AND SURGERY	RETROSPECTIVE	ONCOLOGY	500	SECT	PRE-CONTRAST + DELAYED	NOT REPORTED	60-80 ml	MEASURED	SEPTAL/LEFT VENTRICLE	NONE/FEASIBILITY ONLY	NON-GATED	NO	NO + NOT APPLICABLE	NOT APPLICABLE
**54**	Masataka Suzuki	2021	Japan	Prognostic Impact Of Myocardial Extracellular Volume Fraction Assessment Using Dual- Energy Computed Tomography In Patients Treated With Aortic Valve Replacement For Severe Aortic Stenosis	JOURNAL OF AMERICAN HEART ASSOCIATION	RETROSPECTIVE	AORTIC STENOSIS	95	DECT	PRE-CONTRAST + DELAYED	5'	12 ml	MEASURED	LEFT VENTRICLE	NONE/FEASIBILITY ONLY	GATED	YES	27.80%	ALL-CAUSE DEATH
**55**	Chiara Gnasso	2024	USA, Italy, Germany, Hungary, The Netherlands	ImpaCT Of Reconstruction Parameters On The Accuracy Of Myocardial Extracellular Volume Quantification On A First-Generation, Photon-Counting Detector CT	EUROPEAN RADIOLOGY EXPERIMENTAL	PROSPECTIVE	VARIOUS CARDIAC DISEASE	27	PCCT	DELAYED ONLY	5'	100 ml	NOT REPORTED	NOT REPORTED	CMR	GATED	NO	NO + NOT APPLICABLE	NOT APPLICABLE
**56**	Tetsuya Oguni	2024	Japan	Clinical Characteristics Of Patients With High Extracellular Volume Fraction Evaluated By Cardiac Computed Tomography For Coronary Artery Evaluation	EUROPEAN HEART JOURNAL	RETROSPECTIVE	AMYLOIDOSIS	874	SECT	PRE-CONTRAST + DELAYED	7'	550 mgI/kg	MEASURED	SEPTAL/LEFT VENTRICLE	NONE/FEASIBILITY ONLY	GATED	YES	35.00%	PATHOLOGICAL
**57**	Fabiana Esposito	2024	Italy	CT Images In Follicular Lymphoma: Changes After Treatment Are Predictive Of Cardiac Toxicity In Patients Treated With Anthracycline-Based Or R-B Regimens	CANCERS	RETROSPECTIVE	ONCOLOGY	80	SECT	PRE-CONTRAST + DELAYED	3'-5'	100-120 ml	NOT REPORTED	SEPTAL/LEFT VENTRICLE	NONE/FEASIBILITY ONLY	NOT REPORTED	NO	NO + NOT APPLICABLE	NOT APPLICABLE
**58**	Hye-Jeong Lee	2016	South Korea	Myocardial Extracellular Volume Fraction With Dual-Energy Equilibrium Contrast-Enhanced Cardiac CT In Nonischemic Cardiomyopathy: A Prospective Comparison With Cardiac Mr Imaging	RADIOLOGY	PROSPECTIVE	CARDIOMYOPATHY	30	DECT	DELAYED ONLY	12'	370 mgI/ml x 1.8 ml/kg	MEASURED	LEFT VENTRICLE	CMR	GATED	NO	NO + NOT APPLICABLE	NOT APPLICABLE

**59**	Tsubasa Morioka	2024	Japan	Improvement Of Quantification Of Myocardial Synthetic ECV With Second-Generation Deep Learning Reconstruction	JOURNAL OF CARDIOVASCULAR DEVELOPMENT AND DISEASE	RETROSPECTIVE	VARIOUS CARDIAC DISEASE	40	SECT	PRE-CONTRAST + DELAYED	6'	500 mgI/kg	SYNTHETIC	SEPTAL/LEFT VENTRICLE	NONE/FEASIBILITY ONLY	GATED	NO	NO + NOT APPLICABLE	NOT APPLICABLE
**60**	Shuhei Aoki	2024	Japan	Strong Diagnostic Performance Of Single Energy 256-Row Multidetector Computed Tomography With Deep Learning Image Reconstruction In The Assessment Of Myocardial Fibrosis	INTERNAL MEDICINE	RETROSPECTIVE	VARIOUS CARDIAC DISEASE	50	SECT	PRE-CONTRAST + DELAYED	6'	100 ml	MEASURED	SEPTAL/LEFT VENTRICLE	CMR	GATED	NO	NO + NOT APPLICABLE	NOT APPLICABLE
**61**	Yasutoshi Ohta	2020	Japan	Investigation Of Myocardialextracellular volume Fraction In Heart Failure Patients Using iodine Map With Rapid-KV Switching Dual-Energy CT: Segmental Comparisonwith MRI T1 Mapping	JOURNAL OF CARDIOVASCULAR COMPUTED TOMOGRAPHY	PROSPECTIVE	HEART FAILURE	79	DECT	DELAYED ONLY	7'-8'	370 mgI/ml x 0.9 ml/kg	MEASURED	SEPTAL/LEFT VENTRICLE	CMR	GATED	NO	NO + NOT APPLICABLE	NOT APPLICABLE
**62**	Ahmed Hamdy	2019	Japan	Comparison Of The Different Imaging Time Points In Delayed Phase Cardiac CT For Myocardial Scar Assessment And Extracellular Volume Fraction Estimation In Patients With Old Myocardial Infarction	THE INTERNATIONAL JOURNAL OF CARDIOVASCULAR IMAGING	PROSPECTIVE	CARDIOMYOPATHY	17	SECT	PRE-CONTRAST + DELAYED	3', 5' and 7'	600 mgI/kg	MEASURED	NOT REPORTED	NONE/FEASIBILITY ONLY	GATED	NO	NO + NOT APPLICABLE	NOT APPLICABLE
**63**	Antonio Esposito	2018	Italy	Assessment Of Remote Myocardium Heterogeneity In Patients With Ventricular Tachycardia Using Texture Analysis Of Late Iodine Enhancement (LIE) Cardiac Computed Tomography (CCT) Images	MOLECULAR IMAGING AND BIOLOGY	PROSPECTIVE	CARDIOMYOPATHY	48	SECT	PRE-CONTRAST + DELAYED	10'	0,6 mgI/kg	MEASURED	NOT REPORTED	NONE/FEASIBILITY ONLY	GATED	NO	NO + NOT APPLICABLE	NOT APPLICABLE
**64**	Yasutoshi Ohta	2019	Japan	Quantitative Evaluation Of Non-Ischemic Dilated Cardiomyopathy By Late Iodine Enhancement Using Rapid Kv Switching Dual-Energy Computed Tomography: A Feasibility Study	JOURNAL OF CARDIOVASCULAR COMPUTED TOMOGRAPHY	PROSPECTIVE	CARDIOMYOPATHY	46	DECT	DELAYED ONLY	7'-8'	370 mgI/ml x 0.9 ml/kg	MEASURED	NOT REPORTED	NONE/FEASIBILITY ONLY	GATED	YES	28.20% and 11.18%	DIAGNOSIS OF NON-ISCHEMIC DILATED CARDIOMYOPATHY
**65**	Fumihiko Ogata	2024	Japan	Human Epididymis Protein 4 Is A Useful Predictor Of Post-Operative Prognosis In Patients With Severe Aortic Stenosis	ESC HEART FAILURE	RETROSPECTIVE	AORTIC STENOSIS	55	SECT	NOT REPORTED	NOT REPORTED	NOT REPORTED	NOT REPORTED	NOT REPORTED	NONE/FEASIBILITY ONLY	NOT REPORTED	YES	31.00%	HEART FAILURE HOSPITALIZATION AND DEATH
**66**	van Assen Marly	2019	USA, The Netherlands	Feasibility Of Extracellular Volume Quantification Using Dual-Energy CT	JOURNAL OF CARDIOVASCULAR COMPUTED TOMOGRAPHY	RETROSPECTIVE	VARIOUS CARDIAC DISEASE	42	DECT	PRE-CONTRAST + DELAYED	7'	70 ml	MEASURED	LEFT VENTRICLE	NONE/FEASIBILITY ONLY	GATED	NO	NO + NOT APPLICABLE	NOT APPLICABLE

**67**	Borrazzo Cristian	2020	Italy	Unexpected Increase Of Myocardial Extracellular Volume Fraction In Low Cardiovascular Risk Hiv Patients	TRANSLATIONAL MEDICINE COMMUNICATIONS	RETROSPECTIVE	VARIOUS	53	SECT	PRE-CONTRAST + DELAYED	NOT REPORTED	NOT REPORTED	MEASURED	SEPTAL/LEFT VENTRICLE	NONE/FEASIBILITY ONLY	GATED	NO	NO + NOT APPLICABLE	NOT APPLICABLE
**68**	Yarong Yu	2023	China	Microvascular Myocardial Ischemia In Patients With Diabetes Without Obstructive Coronary Stenosis And Its Association With Angina	KOREAN JOURNAL OF RADIOLOGY	PROSPECTIVE	VARIOUS CARDIAC DISEASE	310	SECT	PRE-CONTRAST + DELAYED	5'	NOT REPORTED	NOT REPORTED	LEFT VENTRICLE	NONE/FEASIBILITY ONLY	NOT REPORTED	NO	NO + NOT APPLICABLE	NOT APPLICABLE
**69**	Klambauer, K.	2025	Switzerland	Photon-Counting Detector Computed Tomography Compared With Cardiac Magnetic Resonance Imaging For Myocardial Extracellular Volume Quantification In Spontaneous Coronary Artery Dissection	NATURE - SCIENTIFIC REPORTS	PROSPECTIVE	VARIOUS CARDIAC DISEASE	13	PCCT	PRE-CONTRAST + DELAYED	5'	60-80 ml	MEASURED	LEFT VENTRICLE	CMR	GATED	YES	28.50%	RELATIVE LESION VOLUMES < 5%
**70**	Chen, Z.X.	2025	China	Dual-Layer Spectral Computed Tomography (CT) Parameters For Identifying Severe Aortic Regurgitation In Aortic Valve Disease Patients	CLINICAL RADIOLOGY	RETROSPECTIVE	VARIOUS CARDIAC DISEASE	53	DECT	DELAYED ONLY	7'	370 mgI/ml x 1,2 ml/kg	MEASURED	LEFT VENTRICLE	NONE/FEASIBILITY ONLY	GATED	YES	29.64%	DIAGNOSIS OF SEVERE AORTIC STENOSIS REGURGITATION
**71**	Mergen, V.	2025	Switzerland	Myocardial Characterization Using Late Enhancement Photon-Counting Detector CT In Ventricular Arrhythmia: Comparison With Electroanatomical Mapping	INSIGHTS INTO IMAGING	RETROSPECTIVE	VARIOUS CARDIAC DISEASE	20	PCCT	PRE-CONTRAST + DELAYED	5'	72-98 ml	MEASURED	NOT REPORTED	ELECTROANATOMICAL MAPPING	GATED	NO	NO + NOT APPLICABLE	NOT APPLICABLE
**72**	Kokawa, T.	2025	Japan	Myocardial Late Enhancement Using Dual-Source Ct: Intraindividual Comparison Of Single-Energy Shuttle And Dual-Energy Acquisition	INSIGHTS INTO IMAGING	PROSPECTIVE	CARDIOMYOPATHY	15	DECT	PRE-CONTRAST + DELAYED	5'	60 ml	MEASURED	SEPTAL/LEFT VENTRICLE	NONE/FEASIBILITY ONLY	GATED	NO	NO + NOT APPLICABLE	NOT APPLICABLE
**73**	Yamaguchi, S.	2025	Japan	Imaging Features Of Wild-Type Transthyretin Amyloid Cardiomyopathy In Gated And Non-Gated Contrast-Enhanced CT	EUROPEAN RADIOLOGY	RETROSPECTIVE	AMYLOIDOSIS	43	SECT	PRE-CONTRAST + DELAYED	7'	550 mg I/kg	NOT REPORTED	SEPTAL MID-VENTRICULAR WALL	NONE/FEASIBILITY ONLY	GATED	YES	55.00%	PROGNOSIS
**74**	Hagar, M.T.	2025	USA	Synthetic Hematocrit-Based Extracellular Volume Quantification From Photon-Counting CT: Validation Against MRI And Systematic Bias Correction	EUROPEAN JOURNAL OF RADIOLOGY	PROSPECTIVE	VARIOUS CARDIAC DISEASE	27	PCCT	PRE-CONTRAST + DELAYED	5'	100 ml	SYNTHETIC	LEFT VENTRICLE	CMR	GATED	NO	NO + NOT APPLICABLE	NOT APPLICABLE
**75**	Popp, S.	2025	Austria	Evaluation Of Extracellular Volume And Coronary Artery Disease In Cardiac Amyloidosis Using Photon-Counting CT	INVESTIGATIVE RADIOLOGY	PROSPECTIVE	AMYLOIDOSIS	30	PCCT	PRE-CONTRAST + DELAYED	5'	62 ml	NOT REPORTED	LEFT VENTRICLE	CMR	GATED	YES	37.00%	DIAGNOSIS

**76**	Aoki, S.	2025	Japan	Extracellular Volume Fraction Analysis On Cardiac Computed Tomography Is Useful For Predicting The Prognosis Of Hypertrophic Cardiomyopathy	JOURNAL OF CARDIOVASCULAR DEVELOPMENT AND DISEASE	RETROSPECTIVE	VARIOUS CARDIAC DISEASE	101	SECT	PRE-CONTRAST + DELAYED	6'	50-110 ml	NOT REPORTED	LEFT VENTRICLE	NONE/FEASIBILITY ONLY	GATED	YES	37.60%	MAJOR ADVERSE CARDIOVASCULAR EVENT
**77**	Belmonte, M.	2025	Belgium	Association Of Cardiac Damage And Computed Tomography–Derived Extracellular Volume In Patients Undergoing Transcatheter Aortic Valve Implantation	CANADIAN JOURNAL OF CARDIOLOGY	PROSPECTIVE	AORTIC STENOSIS	73	SECT	PRE-CONTRAST + DELAYED	8'	100 ml	MEASURED	SEPTAL/LEFT VENTRICLE	NONE/FEASIBILITY ONLY	GATED	YES	32.00%	EVCD PROGRESSION AND STAGES 3-4
**78**	Martuszewski, A.	2025	Poland	Increased Left Ventricular Myocardial Extracellular Volume Assessed By Cardiac Computed Tomography As A Consequence Of Aortic Stenosis And Coexisting Cardiovascular Risk Factors	JOURNAL OF CLINICAL MEDICINE	RETROSPECTIVE	AORTIC STENOSIS	61	SECT	PRE-CONTRAST + DELAYED	3'	70-100 ml	MEASURED	SEPTAL/LEFT VENTRICLE	NONE/FEASIBILITY ONLY	NOT REPORTED	NO	NO + NOT APPLICABLE	NOT APPLICABLE
**79**	Deng, J.	2025	China	Myocardial Extracellular Volume Fraction With Spectral Detector Computed Tomography For Risk Stratification In Non-Ischemic Heart Failure	LA RADIOLOGIA MEDICA	PROSPECTIVE	HEART FAILURE	162	DECT	PRE-CONTRAST + DELAYED	7'	370 mgI/ml x 0,9 ml/kg	MEASURED	LEFT VENTRICLE	CMR	GATED	YES	31.29%	MAJOR ADVERSE CARDIOVASCULAR EVENT
**80**	Lorenzatti, D.A.	2025	USA	Comparison Of Dual-Energy iodine And Standard Subtraction Methods For Myocardial Extracellular Volume Quantification Using Cardiac Computed Tomography	JOURNAL OF CARDIOVASCULAR COMPUTED TOMOGRAPHY	PROSPECTIVE	AORTIC STENOSIS	78	DECT	PRE-CONTRAST + DELAYED	5'	60-160 ml	NOT REPORTED	SEPTAL/LEFT VENTRICLE	NONE/FEASIBILITY ONLY	GATED	NO	NO + NOT APPLICABLE	NOT APPLICABLE
**81**	Gkizas, C.,	2025	France	Photon-Counting CT-Derived Extracellular Volume In Acute Myocarditis: Comparison With Cardiac MRI	DIAGNOSTIC AND INTERVENTIONAL IMAGING	RETROSPECTIVE	MYOCARDITIS	32	PCCT	DELAYED ONLY	5'	50 ml	MEASURED	NOT REPORTED	CMR	GATED	YES	26.90%	DIAGNOSIS
**82**	Kojima, T.	2025	Japan	Clinical Utility Of Non-Gated 4-Min Delayed Dual-Energy CT For Myocardial Extracellular Volume Quantification	BRITISH JOURNAL OF RADIOLOGY	RETROSPECTIVE	VARIOUS CARDIAC DISEASE	29	DECT	DELAYED ONLY	4'	60-100 ml	NOT REPORTED	LEFT VENTRICLE	NONE/FEASIBILITY ONLY	NON-GATED	NO	NO + NOT APPLICABLE	NOT APPLICABLE
**83**	Kagawa, Y.	2025	Japan	Myocardial Extracellular Volume Fraction Estimations Using Late Enhancement CT In Patients With Atrial Fibrillation: A Comparative Study With Cardiac MR	THE INTERNATIONAL JOURNAL OF CARDIOVASCULAR IMAGING	RETROSPECTIVE	VARIOUS CARDIAC DISEASE	42	SECT	PRE-CONTRAST + DELAYED	5'	NOT REPORTED	MEASURED	LEFT VENTRICLE	CMR	GATED	NO	NO + NOT APPLICABLE	NOT APPLICABLE
**84**	Wu, H.	2025	China	Quantitative Assessment Of Early Changes In Myocardial Extracellular Volume During Postoperative Adjuvant Chemotherapy In	QUANTITATIVE IMAGING IN MEDICINE AND SURGERY	PROSPECTIVE	ONCOLOGY	40	DECT	DELAYED ONLY	7'	50-90 ml	NOT REPORTED	LEFT VENTRICLE	NONE/FEASIBILITY ONLY	NOT REPORTED	NO	NO + NOT APPLICABLE	NOT APPLICABLE

				Patients With Breast Cancer Via Dual-Layer Spectral Detector Computed Tomography: A Cohort Study															
**85**	Lisi, C.	2025	Switzerland	Relationship Between Myocardial Strain And Extracellular Volume: Exploratory Study In Patients With Severe Aortic Stenosis Undergoing Photon-Counting Detector CT	DIAGNOSTICS	RETROSPECTIVE	AORTIC STENOSIS	77	PCCT	PRE-CONTRAST + DELAYED	5'	60-80 ml	MEASURED	NOT REPORTED	NONE/FEASIBILITY ONLY	GATED	NO	NO + NOT APPLICABLE	NOT APPLICABLE
**86**	Yuan, J.,	2025	China	The Impact Of Lipoprotein(A) Level On Cardiac Pathologies In Diabetes: A Cardiac Ct Study	EUROPEAN RADIOLOGY	PROSPECTIVE	VARIOUS	207	SECT	PRE-CONTRAST + DELAYED	5'	NOT REPORTED	NOT REPORTED	NOT REPORTED	NONE/FEASIBILITY ONLY	GATED	NO	NO + NOT APPLICABLE	NOT APPLICABLE
**87**	Franceschi, P.	2025	Italy	Ultrafast Non-ECG-Gated Cardiac Spectral CT Scanning For Myocardial Late iodine Enhancement Assessment: A Feasibility Study	CARDIAC RADIOLOGY	RETROSPECTIVE	VARIOUS CARDIAC DISEASE	51	DECT	PRE-CONTRAST + DELAYED	6'-7'	<100 ml	MEASURED	SEPTAL/LEFT VENTRICLE	NONE/FEASIBILITY ONLY	NON-GATED	NO	NO + NOT APPLICABLE	NOT APPLICABLE
**88**	Gkizas, C.	2025	France	Photon-Counting CT Myocardial Extracellular Volume: A Non-Invasive Biomarker For Fibrosis In Patients With Hypertrophic Cardiomyopathy	DIAGNOSTIC AND INTERVENTIONAL IMAGING	RETROSPECTIVE	CARDIOMYOPATHY	30	PCCT	DELAYED ONLY	5'	50 ml	MEASURED	NOT REPORTED	NONE/FEASIBILITY ONLY	GATED	YES	33.40%	DIAGNOSIS OF FIBROSIS
**89**	Bao, S.	2025	China	Dual-Energy Based Myocardial Extracellular Volume Quantification With Photon-Counting Detector And Energy-Integrating Detector Dual-Source Computed Tomography: Comparison With Cardiac Magnetic Resonance	EUROPEAN RADIOLOGY	RETROSPECTIVE	VARIOUS CARDIAC DISEASE	80	PCCT	DELAYED ONLY	5'	370 mgI/ml x 0,8 ml/kg	MEASURED	LEFT VENTRICLE	CMR	GATED	NO	NO + NOT APPLICABLE	NOT APPLICABLE
**90**	Kokawa, T.	2025	Japan	ImpaCT Of Contrast Medium Volume On Myocardial Extracellular Volume Measurement Accuracy In First-Generation Photon-Counting Detector CT	THE INTERNATIONAL JOURNAL OF CARDIOVASCULAR IMAGING	PROSPECTIVE	VARIOUS CARDIAC DISEASE	40	PCCT	PRE-CONTRAST + DELAYED	5'	100 ml	MEASURED	SEPTAL/LEFT VENTRICLE	NONE/FEASIBILITY ONLY	NOT REPORTED	NO	NO + NOT APPLICABLE	NOT APPLICABLE
**91**	Klambauer, K.	2025	Switzerland	Myocardial Late Enhancement With Photon-Counting Detector CT In Spontaneous Coronary Artery Dissection Prospective Comparison With Cardiac MRI	INVESTIGATIVE RADIOLOGY	PROSPECTIVE	VARIOUS CARDIAC DISEASE	17	PCCT	PRE-CONTRAST + DELAYED	5'	60-80 ml	MEASURED	NOT REPORTED	CMR	GATED	NO	NO + NOT APPLICABLE	NOT APPLICABLE
**92**	Yashima, S.	2024	Japan	Extracellular Volume By Computed Tomography Is Useful For Prediction Of Prognosis In Dilated Cardiomyopathy Cases With Heart Failure	JOURNAL OF CARDIOVASCULAR DEVELOPMENT AND DISEASE	RETROSPECTIVE	CARDIOMYOPATHY	101	SECT	PRE-CONTRAST + DELAYED	6'	45-100 ml	NOT REPORTED	SEPTAL/LEFT VENTRICLE	NONE/FEASIBILITY ONLY	GATED	YES	32.30%	MAJOR ADVERSE CARDIOVASCULAR EVENT

				With Reduced Ejection Fraction															
**93**	Tu, C.	2024	China	Longitudinal Evaluation Of Coronary Arteries And Myocardium In Breast Cancer Using Coronary Computed Tomographic Angiography	JOURNAL OF THE AMERICAN COLLEGE OF CARDIOLOGY	PROSPECTIVE	ONCOLOGY	139	DECT	PRE-CONTRAST + DELAYED	5'	320 mg I/ml x 1,5 ml/kg	MEASURED	NOT REPORTED	NONE/FEASIBILITY ONLY	GATED	NO	NO + NOT APPLICABLE	NOT APPLICABLE
**94**	Hayashi, H.	2023	Japan	Myocardial Extracellular Volume Quantification In Cardiac Amyloidosis: A Comparative Study Between Cardiac Computed Tomography And Magnetic Resonance Imaging	EUROPEAN RADIOLOGY	RETROSPECTIVE	AMYLOIDOSIS	31	SECT	PRE-CONTRAST + DELAYED	NOT REPORTED	550 mg I/kg	MEASURED	LEFT VENTRICLE	CMR	GATED	NO	NO + NOT APPLICABLE	NOT APPLICABLE
**95**	Baggiano, A.	2023	Italy	Quantification Of Extracellular Volume With Cardiac Computed Tomography In Patients With Dilated Cardiomyopathy	JOURNAL OF CARDIOVASCULAR COMPUTED TOMOGRAPHY	PROSPECTIVE	CARDIOMYOPATHY	39	SECT	PRE-CONTRAST + DELAYED	8'	80 ml	MEASURED	LEFT VENTRICLE	CMR	GATED	NO	NO + NOT APPLICABLE	NOT APPLICABLE
**96**	Koike, H.	2023	USA	Importance Of Imaging-Acquisition Protocol And Post-Processing Analysis For Extracellular Volume Fraction Assessment By Computed Tomography	JOURNAL OF CARDIOVASCULAR COMPUTED TOMOGRAPHY	PROSPECTIVE	AORTIC STENOSIS	134	SECT	PRE-CONTRAST + DELAYED	3'	100 ml	NOT REPORTED	LEFT VENTRICLE	NONE/FEASIBILITY ONLY	GATED	NO	NO + NOT APPLICABLE	NOT APPLICABLE
**97**	Nishikawa, Y.	2023	Japan	A New Composite Indicator Consisting Of Left Ventricular Extracellular Volume, N-Terminal Fragment Of B-Type Natriuretic Peptide, And Left Ventricular End-Diastolic Volume Is Useful For Predicting Reverse Remofeling After Catheter Ablation For Atrial Fibrillation	HEART AND VESSELS	RETROSPECTIVE	VARIOUS CARDIAC DISEASE	33	SECT	PRE-CONTRAST + DELAYED	6'	50-110 ml	NOT REPORTED	LEFT VENTRICLE	NONE/FEASIBILITY ONLY	GATED	YES	37.73%	PREDICTION OF REVERSE REMODELING
**98**	Abadia, A.F.	2022	Germany	Automated Dual-Energy Computed Tomography-Based Extracellular Volume Estimation For Myocardial Characterization In Patients With Ischemic And Nonischemic Cardiomyopathy	JOURNAL OF THORACIC IMAGING	RETROSPECTIVE	CARDIOMYOPATHY	9	DECT	DELAYED ONLY	7'	70 ml	MEASURED	LEFT VENTRICLE	NONE/FEASIBILITY ONLY	GATED	NO	NO + NOT APPLICABLE	NOT APPLICABLE
**99**	Hayashi, H.	2022	Japan	Myocardial Extracellular Volume Quantification By Cardiac CT In Pulmonary Hypertension: Comparison With Cardiac MRI	EUROPEAN JOURNAL OF RADIOLOGY	RETROSPECTIVE	VARIOUS	20	DECT	DELAYED ONLY	7'	550 mgI/kg	NOT REPORTED	NOT REPORTED	CMR	GATED	NO	NO + NOT APPLICABLE	NOT APPLICABLE

**100**	Shao, J.	2022	China	Measurement Of Myocardial Extracellular Volume Using Cardiac Dual-Energy Computed Tomography In Patients With Ischaemic Cardiomyopathy: A Comparison Of Different Methofs	THE INTERNATIONAL JOURNAL OF CARDIOVASCULAR IMAGING	RETROSPECTIVE	CARDIOMYOPATHY	57	DECT	DELAYED ONLY	7'	50 ml	MEASURED	LEFT VENTRICLE	NONE/FEASIBILITY ONLY	GATED	NO	NO + NOT APPLICABLE	NOT APPLICABLE
**101**	Qi, R.	2022	China	Measurement Of Myocardial Extracellular Volume Fraction In Patients With Heart Failure With Preserved Ejection Fraction Using Dual-Energy Computed Tomography	EUROPEAN RADIOLOGY	RETROSPECTIVE	HEART FAILURE	112	DECT	DELAYED ONLY	7'	50 ml	NOT REPORTED	LEFT VENTRICLE	NONE/FEASIBILITY ONLY	GATED	NO	NO + NOT APPLICABLE	NOT APPLICABLE
**102**	Rodriguez-Granillo, G.A.	2022	Argentina	Hyperacute Incidental Late Myocardial Enhancement In Ischemic Stroke Using Chest Spectral Ct: Relationship With Etiology	REVIEWS IN CARDIOVASCULAR MEDICINE	PROSPECTIVE	VARIOUS	80	DECT	DELAYED ONLY	5'	NOT REPORTED	MEASURED	SEPTAL/LEFT VENTRICLE	NONE/FEASIBILITY ONLY	NON-GATED	NO	NO + NOT APPLICABLE	NOT APPLICABLE
**103**	Shiyovich, A.	2022	Israel	Relation Of Serum Albumin Levels To Myocardial Extracellular Volume In Patients With Severe Aortic Stenosis	THE AMERICAN JOURNAL OF CARDIOLOGY	PROSPECTIVE	AORTIC STENOSIS	68	SECT	PRE-CONTRAST + DELAYED	7'	50-60 ml	NOT REPORTED	LEFT VENTRICLE	NONE/FEASIBILITY ONLY	GATED	NO	NO + NOT APPLICABLE	NOT APPLICABLE
**104**	Capra, D.	2020	Italy	Computed Tomography-Derived Myocardial Extracellular Volume: An Early Biomarker Of Cardiotoxicity In Esophageal Cancer Patients Undergoing Radiation Therapy	INSIGHTS INTO IMAGING	RETROSPECTIVE	ONCOLOGY	21	SECT	ATTENUATION-BASED	NOT REPORTED	NOT REPORTED	MEASURED	SEPTAL/LEFT VENTRICLE	NONE/FEASIBILITY ONLY	NON-GATED	NO	NO + NOT APPLICABLE	NOT APPLICABLE
**105**	Monti, C.B.	2020	Italy	Assessment Of Myocardial Extracellular Volume On Bofy Computed Tomography In Breast Cancer Patients Treated With Anthracyclines	QUANTITATIVE IMAGING IN MEDICINE AND SURGERY	RETROSPECTIVE	ONCOLOGY	32	SECT	PRE-CONTRAST + DELAYED	7'	370 mgI/ml x 1,2 ml/kg	NOT REPORTED	SEPTAL/LEFT VENTRICLE	NONE/FEASIBILITY ONLY	NON-GATED	NO	NO + NOT APPLICABLE	NOT APPLICABLE
**106**	Ohta, Y.	2017	Japan	Measurement Of Myocardial Extracellular Volume Fraction From iodine Density Images Using Single-Source, Dual-Energy Computed Tomography: A Feasibility Study	JOURNAL OF COMPUTER ASSISTED TOMOGRAPHY	PROSPECTIVE	VARIOUS CARDIAC DISEASE	40	DECT	PRE-CONTRAST + DELAYED	7'	370 mgI/ml x 0,9 ml/kg	MEASURED	SEPTAL/LEFT VENTRICLE	NONE/FEASIBILITY ONLY	GATED	NO	NO + NOT APPLICABLE	NOT APPLICABLE
**107**	Treibel, T.A.	2017	UK	Automatic Quantification Of The Myocardial Extracellular Volume By Cardiac Computed Tomography: Synthetic ECV By CCT	JOURNAL OF CARDIOVASCULAR COMPUTED TOMOGRAPHY	RETROSPECTIVE	VARIOUS CARDIAC DISEASE	40	SECT	PRE-CONTRAST + DELAYED	5'	300 mgI/kg x 1 ml/kg	SYNTHETIC	SEPTAL/LEFT VENTRICLE	NONE/FEASIBILITY ONLY	GATED	NO	NO + NOT APPLICABLE	NOT APPLICABLE
**108**	Sayama, K.	2025	Japan	Increasing Extracellular Volume Fraction On Coronary Cta In Patients With Coronary Microvascular Dysfunction	CIRCULATION CARDIOVASCULAR IMAGING	RETROSPECTIVE	VARIOUS CARDIAC DISEASE	57	SECT	PRE-CONTRAST + DELAYED	7'	80 ml	MEASURED	LEFT VENTRICLE	NONE/FEASIBILITY ONLY	GATED	NO	NO + NOT APPLICABLE	NOT APPLICABLE

**109**	Zhang, X.	2025	China	CT-Derived Myocardial Extracellular Volume Predict Major Adverse Cardiovascular Events In Patients With Prostate Cancer	EUROPEAN JOURNAL OF RADIOLOGY	RETROSPECTIVE	ONCOLOGY	180	SECT	PRE-CONTRAST + DELAYED	NOT REPORTED	NOT REPORTED	MEASURED	SEPTAL/LEFT VENTRICLE	NONE/FEASIBILITY ONLY	NOT REPORTED	YES	23.45%, 27.93%, 27.07%, 27.35%	MAJOR ADVERSE CARDIOVASCULAR EVENT
**110**	McShane, JN	2025	Canada	CT-Derived ECV In Severe Aortic Stenosis: Prognosticator And Screening Test For Co-Existent Transthyretin Cardiac Amyloidosis	AMERICAN HEART JOURNAL PLUS: CARDIOLOGY RESEARCH AND PRACTICE	PROSPECTIVE	AORTIC STENOSIS	161	SECT	PRE-CONTRAST + DELAYED	5'	50 ml	NOT REPORTED	SEPTAL/LEFT VENTRICLE	NONE/FEASIBILITY ONLY	GATED	YES	31.00%	DIAGNOSIS
**111**	Saberwal, B	2025	UK	Myocardial Extracellular Volume Fraction By Computed Tomography Vs. Cardiovascular Magnetic Resonance Imaging In Patients With Stable Chest Pain	EUROPEAN HEART JOURNAL	PROSPECTIVE	VARIOUS	137	SECT	PRE-CONTRAST + DELAYED	5'	130 ml	MEASURED	NOT REPORTED	CMR	GATED	NO	NO + NOT APPLICABLE	NOT APPLICABLE
**112**	Kidoh, M	2025	Japan	CT-Derived Extracellular Volume Fraction In Aortic Stenosis, Cardiac Amyloidosis, And Dual Pathology	EUROPEAN HEART JOURNAL	RETROSPECTIVE	AORTIC STENOSIS AND AMYLOIDOSIS	138	SECT	PRE-CONTRAST + DELAYED	7'	550 mgI/kg	MEASURED	SEPTAL/LEFT VENTRICLE	SCINTIGRAPHY	NOT REPORTED	NO	NO + NOT APPLICABLE	NOT APPLICABLE
**113**	Malhotra, P	2024	USA	Predictive Value Of CTA-Derived Extracellular Volume For Pacemaker Implantation Post-TAVR In Low-Flow Low-Gradient Aortic Stenosis	JOURNAL OF THE AMERICAN COLLEGE OF CARDIOLOGY	RETROSPECTIVE	AORTIC STENOSIS	122	SECT	PRE-CONTRAST + DELAYED	3'-5'	NOT REPORTED	MEASURED	NOT REPORTED	NONE/FEASIBILITY ONLY	GATED	NO	NO + NOT APPLICABLE	NOT APPLICABLE

Table S2 Overall distribution of studies across pathologies

PATHOLOGY	N.	%
**AMYLOIDOSIS**	14	12.4%
**AORTIC STENOSIS**	21	18.6%
**CARDIOMYOPATHY**	14	12.4%
**HEART FAILURE**	7	6.2%
**MYOCARDITIS**	3	2.7%
**ONCOLOGY**	13	11.5%
**VARIOUS**	8	7.1%
**VARIOUS CARDIAC DISEASE**	33	29.2%
**TOT.**	**113**	**100.0%**

Fig. S1 Observational study design distribution

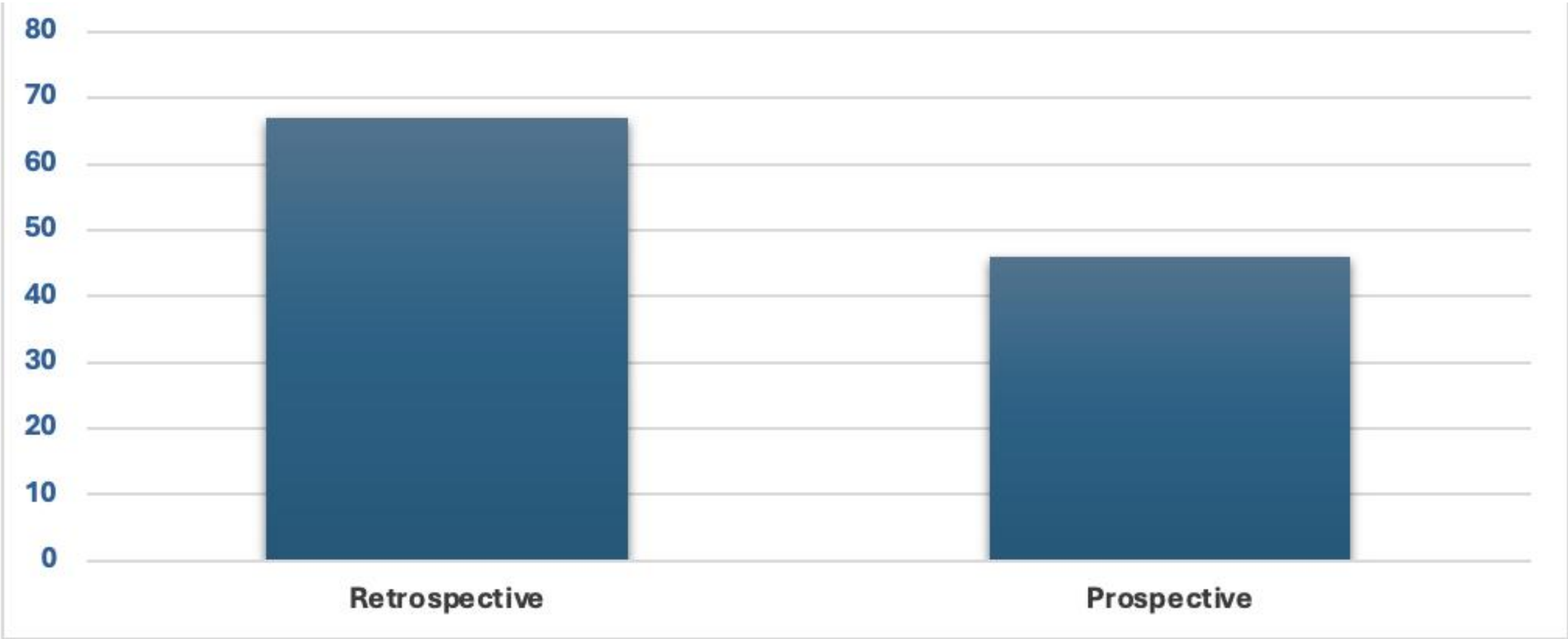

Supplementary Figure Legends

Fig. S1 - Observational study design distribution.

Supplementary Table Legends

Table S1 - Data extraction.

Table S2 - Overall distribution of studies across pathologies.
