# Supplementary material for: CT-derived myocardial extracellular volume: current evidence, clinical utility, and the emerging role of photon-counting CT—a systematic review
Source: Eur Radiol Exp. 2026 Sep 22;10:142. doi: 10.1186/s41747-026-00808-w (PMC13598009; doi:10.1186/s41747-026-00808-w)
Supplement: Supplementary file 2 [file 41747_2026_808_MOESM2_ESM.docx]

**ADDITIONAL FILE**

**Search strings**

**PubMed**

(("extracellular volume" OR "ECV" OR "extracellular volume fraction") AND ("heart" OR "myocardium" OR "cardiac") AND ("computed tomography" OR "CT" OR "dual-energy CT" OR "DECT" OR "cardiac CT" OR "photon-counting CT" OR "photon counting CT" OR "photon-counting detector")) AND ("2015/01/01"[Date - Publication]: "3000" [Date - Publication]) NOT ("animals"[Title/Abstract] OR "in vitro"[Title/Abstract] OR "review"[Publication Type])

**Scopus**

TITLE-ABS ("extracellular volume" OR "extracellular volume fraction" OR ECV) AND TITLE-ABS ("heart" OR myocard* OR cardiac) AND TITLE-ABS ("computed tomography" OR "cardiac CT" OR "dual-energy CT" OR "dual energy CT" OR DECT OR "photon-counting CT" OR "photon counting CT" OR "photon-counting detector") AND NOT TITLE-ABS ("in vitro") AND NOT DOCTYPE(review)

**Embase**

('extracellular volume':ab,ti OR 'extracellular volume fraction':ab,ti OR ecv:ab,ti) AND ('heart':ab,ti OR myocard*:ab,ti OR cardiac:ab,ti) AND ('computed tomography':ab,ti OR 'cardiac ct':ab,ti OR 'dual-energy ct':ab,ti OR 'dual energy ct':ab,ti OR dect:ab,ti OR 'photon-counting ct':ab,ti OR 'photon counting ct':ab,ti OR 'photon-counting detector':ab,ti) NOT ('in vitro study'/exp) NOT 'review'/it NOT 'conference abstract'/it
