# Supplementary material for: CT-derived myocardial extracellular volume: current evidence, clinical utility, and the emerging role of photon-counting CT—a systematic review
Source: Eur Radiol Exp. 2026 Sep 22;10:142. doi: 10.1186/s41747-026-00808-w (PMC13598009; doi:10.1186/s41747-026-00808-w)
Supplement: Supplementary file 3 [file 41747_2026_808_MOESM3_ESM.docx]

**FULL LIST OF INCLUDED ARTICLES**[1–113]

1.	Monti CB, Zanardo M, Bosetti T et al (2020) Assessment of myocardial extracellular volume on body computed tomography in breast cancer patients treated with anthracyclines. Quant Imaging Med Surg 10:934–944. https://doi.org/10.21037/qims.2020.04.05

2.	Yashima S, Takaoka H, Ota J et al (2024) Extracellular volume by computed tomography is useful for prediction of prognosis in dilated cardiomyopathy cases with heart failure with reduced ejection fraction. J Cardiovasc Dev Dis 11:399. https://doi.org/10.3390/jcdd11120399

3.	Borrazzo C, d’Ettorre G, Ceccarelli G et al (2020) Unexpected increase of myocardial extracellular volume fraction in low cardiovascular risk hiv patients. Transl Med Commun 5:24. https://doi.org/10.1186/s41231-020-00077-8

4.	Aoki S, Takaoka H, Kanaeda T et al (2025) Extracellular volume fraction analysis on cardiac computed tomography is useful for predicting the prognosis of hypertrophic cardiomyopathy. J Cardiovasc Dev Dis 12:372. https://doi.org/10.3390/jcdd12090372

5.	Yu Y, Yang W, Dai X et al (2023) Microvascular myocardial ischemia in patients with diabetes without obstructive coronary stenosis and its association with angina. Korean J Radiol 24:1081–1092. https://doi.org/10.3348/kjr.2023.0297

6.	van Assen M, De Cecco CN, Sahbaee P et al (2019) Feasibility of extracellular volume quantification using dual-energy ct. J Cardiovasc Comput Tomogr 13:81–84. https://doi.org/10.1016/j.jcct.2018.10.011

7.	Wu H, Huang Y, Yang M et al (2024) Quantitative assessment of early changes in myocardial extracellular volume during postoperative adjuvant chemotherapy in patients with breast cancer via dual-layer spectral detector computed tomography: a cohort study. Quant Imaging Med Surg 15:404–416. https://doi.org/10.21037/qims-24-777

8.	Lisi C, Mergen V, Moser LJ et al (2025) Relationship between myocardial strain and extracellular volume: exploratory study in patients with severe aortic stenosis undergoing photon-counting detector ct. Diagnostics 15:224. https://doi.org/10.3390/diagnostics15020224

9.	Martuszewski A, Paluszkiewicz P, Poręba R, Gać P (2025) Increased left ventricular myocardial extracellular volume assessed by cardiac computed tomography as a consequence of aortic stenosis and coexisting cardiovascular risk factors. J Clin Med 14:4435. https://doi.org/10.3390/jcm14134435

10.	Capra D, Monti CB, Luporini AG et al (2020) Computed tomography-derived myocardial extracellular volume: an early biomarker of cardiotoxicity in esophageal cancer patients undergoing radiation therapy. Insights Imaging 11:120. https://doi.org/10.1186/s13244-020-00922-2

11.	Kidoh M, Oda S, Tabata N et al (2025) CT-derived extracellular volume fraction in aortic stenosis, cardiac amyloidosis, and dual pathology. Eur Heart J Cardiovasc Imaging 26:509–517. https://doi.org/10.1093/ehjci/jeae320

12.	Qi R-X, Jiang J-S, Shao J et al (2022) Measurement of myocardial extracellular volume fraction in patients with heart failure with preserved ejection fraction using dual-energy computed tomography. Eur Radiol 32:4253–4263. https://doi.org/10.1007/s00330-021-08514-4

13.	Baggiano A, Conte E, Spiritigliozzi L et al (2023) Quantification of extracellular volume with cardiac computed tomography in patients with dilated cardiomyopathy. J Cardiovasc Comput Tomogr 17:261–268. https://doi.org/10.1016/j.jcct.2023.04.005

14.	McShane JN, Tavoosi A, El Mais H et al (2025) CT derived ecv in severe aortic stenosis: prognosticator and screening test for co-existent transthyretin cardiac amyloidosis. American Heart Journal Plus: Cardiology Research and Practice 56:100575. https://doi.org/10.1016/j.ahjo.2025.100575

15.	Mergen V, Reiner MF, Klambauer K et al (2025) Myocardial characterization using late enhancement photon-counting detector ct in ventricular arrhythmia: comparison with electroanatomical mapping. Insights Imaging 16:187. https://doi.org/10.1186/s13244-025-02069-4

16.	Gkizas C, Longere B, Sliwicka O et al (2025) Photon-counting ct-derived extracellular volume in acute myocarditis: comparison with cardiac mri. Diagn Interv Imaging 106:255–263. https://doi.org/10.1016/j.diii.2025.03.001

17.	Deng J, Wang Y, Qi T et al (2025) Myocardial extracellular volume fraction with spectral detector computed tomography for risk stratification in non-ischemic heart failure. Radiol Med 130:1092–1104. https://doi.org/10.1007/s11547-025-02002-1

18.	Yamaguchi S, Oda S, Kidoh M et al (2025) Imaging features of wild-type transthyretin amyloid cardiomyopathy in gated and non-gated contrast-enhanced ct. Eur Radiol 35:7064–7073. https://doi.org/10.1007/s00330-025-11631-z

19.	Ohta Y, Kitao S, Watanabe T et al (2017) Measurement of myocardial extracellular volume fraction from iodine density images using single-source, dual-energy computed tomography: a feasibility study. J Comput Assist Tomogr 41:750–756. https://doi.org/10.1097/RCT.0000000000000587

20.	Nishikawa Y, Takaoka H, Kanaeda T et al (2023) A new composite indicator consisting of left ventricular extracellular volume, n-terminal fragment of b-type natriuretic peptide, and left ventricular end-diastolic volume is useful for predicting reverse remodeling after catheter ablation for atrial fibrillation. Heart Vessels 38:721–730. https://doi.org/10.1007/s00380-022-02220-x

21.	Saberwal B, Patel K, Klotz E et al (2025) Myocardial extracellular volume fraction by computed tomography vs. cardiovascular magnetic resonance imaging in patients with stable chest pain. European Heart Journal - Imaging Methods and Practice 3:qyaf019. https://doi.org/10.1093/ehjimp/qyaf019

22.	Koike H, Cheng VY, Lesser A et al (2023) Importance of imaging-acquisition protocol and post-processing analysis for extracellular volume fraction assessment by computed tomography. J Cardiovasc Comput Tomogr 17:222–225. https://doi.org/10.1016/j.jcct.2023.02.007

23.	Kokawa T, Nakamura S, Takafuji M, Yamazaki A, Sakuma H, Kitagawa K (2025) Impact of contrast medium volume on myocardial extracellular volume measurement accuracy in first-generation photon-counting detector ct. Int J Cardiovasc Imaging 42:423–434. https://doi.org/10.1007/s10554-025-03437-3

24.	Hayashi H, Oda S, Kidoh M et al (2024) Myocardial extracellular volume quantification in cardiac amyloidosis: a comparative study between cardiac computed tomography and magnetic resonance imaging. Eur Radiol 34:1016–1025. https://doi.org/10.1007/s00330-023-10129-w

25.	Klambauer K, Klotz E, Moser LJ et al (2026) Myocardial late enhancement with photon-counting detector ct in spontaneous coronary artery dissection: prospective comparison with cardiac mri. Invest Radiol 61:32–40. https://doi.org/10.1097/RLI.0000000000001203

26.	Abadia AF, Aquino GJ, Schoepf UJ et al (2022) Automated dual-energy computed tomography-based extracellular volume estimation for myocardial characterization in patients with ischemic and nonischemic cardiomyopathy. J Thorac Imaging 37:307–314. https://doi.org/10.1097/RTI.0000000000000656

27.	Kokawa T, Kitagawa K, Nakamura S, Takafuji M, Oya T, Sakuma H (2025) Myocardial late enhancement using dual-source ct: intraindividual comparison of single-energy shuttle and dual-energy acquisition. Insights Imaging 16:64. https://doi.org/10.1186/s13244-025-01944-4

28.	Shao J, Jiang J-S, Wang X-Y et al (2022) Measurement of myocardial extracellular volume using cardiac dual-energy computed tomography in patients with ischaemic cardiomyopathy: a comparison of different methods. Int J Cardiovasc Imaging 38:1591–1600. https://doi.org/10.1007/s10554-022-02532-z

29.	Yuan J, Ding X, Yang W et al (2025) The impact of lipoprotein(a) level on cardiac pathologies in diabetes: a cardiac ct study. Eur Radiol 35:220–231. https://doi.org/10.1007/s00330-024-10903-4

30.	Gkizas C, Longere B, Bechrouri S et al (2025) Photon-counting ct myocardial extracellular volume: a non-invasive biomarker for fibrosis in patients with hypertrophic cardiomyopathy. Diagn Interv Imaging 107:17–24. https://doi.org/10.1016/j.diii.2025.09.001

31.	Belmonte M, Paolisso P, de Oliveira EK et al (2025) Association of cardiac damage and computed tomography derived extracellular volume in patients undergoing transcatheter aortic valve implantation. Canadian Journal of Cardiology 41:1546–1557. https://doi.org/10.1016/j.cjca.2025.02.031

32.	Rodriguez-Granillo GA, Cirio JJ, Ciardi C et al (2022) Hyperacute incidental late myocardial enhancement in ischemic stroke using chest spectral ct: relationship with etiology. Rev Cardiovasc Med 23:93. https://doi.org/10.31083/j.rcm2303093

33.	Kojima T, Yamasaki Y, Nishigake D et al (2025) Clinical utility of non-gated 4-min delayed dual-energy ct for myocardial extracellular volume quantification. British Journal of Radiology 98:600–606. https://doi.org/10.1093/bjr/tqaf022

34.	Lorenzatti DA, Filtz A, Piña P et al (2025) Comparison of dual-energy iodine and standard subtraction methods for myocardial extracellular volume quantification using cardiac computed tomography. J Cardiovasc Comput Tomogr 19:483–487. https://doi.org/10.1016/j.jcct.2025.03.009

35.	Shiyovich A, Plakht Y, Hammer Y et al (2022) Relation of serum albumin levels to myocardial extracellular volume in patients with severe aortic stenosis. American Journal of Cardiology 163:71–76. https://doi.org/10.1016/j.amjcard.2021.09.044

36.	Bao S, Zhou M, Wang M, Xu Z, Yan F, Yang W (2025) Dual-energy based myocardial extracellular volume quantification with photon-counting detector and energy-integrating detector dual-source computed tomography: comparison with cardiac magnetic resonance. Eur Radiol 36:1989–2000. https://doi.org/10.1007/s00330-025-11942-1

37.	Treibel TA, Fontana M, Steeden JA et al (2017) Automatic quantification of the myocardial extracellular volume by cardiac computed tomography: synthetic ecv by cct. J Cardiovasc Comput Tomogr 11:221–226. https://doi.org/https://doi.org/10.1016/j.jcct.2017.02.006

38.	Popp S, Beitzke D, Strassl A et al (2025) Evaluation of extracellular volume and coronary artery disease in cardiac amyloidosis using photon-counting ct. Invest Radiol 60:698–707. https://doi.org/10.1097/RLI.0000000000001198

39.	Pankaj M, Donghee H, Billy C et al (2024) Predictive value of cta-derived extracellular volume for pacemaker implantation post-tavr in low-flow low-gradient aortic stenosis. JACC Cardiovasc Imaging 17:563–565. https://doi.org/10.1016/j.jcmg.2023.11.012

40.	Zhang X, Shen H, Xu Q et al (2025) CT derived myocardial extracellular volume predict major adverse cardiovascular events in patients with prostate cancer. Eur J Radiol 192:112371. https://doi.org/10.1016/j.ejrad.2025.112371

41.	Chunrong T, Hesong S, Xiaoqin L et al (2024) Longitudinal evaluation of coronary arteries and myocardium in breast cancer using coronary computed tomographic angiography. JACC Cardiovasc Imaging 17:1335–1347. https://doi.org/10.1016/j.jcmg.2024.05.017

42.	Sayama K, Kanaji Y, Usui E et al (2025) Increasing extracellular volume fraction on coronary cta in patients with coronary microvascular dysfunction. Circ Cardiovasc Imaging 18:e019082. https://doi.org/10.1161/CIRCIMAGING.125.018368

43.	Franceschi P, Sportoletti C, Rasciti E et al (2025) Ultrafast non-ecg-gated cardiac spectral ct scanning for myocardial late iodine enhancement assessment: a feasibility study. Radiol Med 131:428–439. https://doi.org/10.1007/s11547-025-02114-8

44.	Hayashi H, Oda S, Emoto T et al (2022) Myocardial extracellular volume quantification by cardiac ct in pulmonary hypertension: comparison with cardiac mri. Eur J Radiol 153:110386. https://doi.org/10.1016/j.ejrad.2022.110386

45.	Hagar MT, Moore WG, Vecsey-Nagy M et al (2025) Synthetic hematocrit-based extracellular volume quantification from photon-counting ct: validation against mri and systematic bias correction. Eur J Radiol 191:112321. https://doi.org/10.1016/j.ejrad.2025.112321

46.	Kagawa Y, Takafuji M, Fujita S et al (2025) Myocardial extracellular volume fraction estimations using late enhancement ct in patients with atrial fibrillation: a comparative study with cardiac mr. Int J Cardiovasc Imaging 41:419–426. https://doi.org/10.1007/s10554-024-03316-3

47.	Chen ZX, Xu J, Tian W et al (2025) Dual-layer spectral computed tomography (ct) parameters for identifying severe aortic regurgitation in aortic valve disease patients. Clin Radiol 91:107067. https://doi.org/10.1016/j.crad.2025.107067

48.	Klambauer K, Biondo A, Klotz E et al (2025) Photon-counting detector computed tomography compared with cardiac magnetic resonance imaging for myocardial extracellular volume quantification in spontaneous coronary artery dissection. Sci Rep 15:39923. https://doi.org/10.1038/s41598-025-23777-3

49.	Yashima S, Takaoka H, Iwahana T et al (2023) Evaluation of extracellular volume by computed tomography is useful for prediction of prognosis in dilated cardiomyopathy. Heart Vessels 38:185–194. https://doi.org/10.1007/s00380-022-02154-4

50.	Liu P, Lin L, Xu C et al (2022) Quantitative analysis of late iodine enhancement using dual-layer spectral detector computed tomography: comparison with magnetic resonance imaging. Quant Imaging Med Surg 12:310–320. https://doi.org/10.21037/qims-21-344

51.	Kadoya Y, Omaygenc MO, Chow B, Small GR (2024) Reproducibility of myocardial extracellular volume quantification using dual-energy computed tomography in patients with cardiac amyloidosis. J Cardiovasc Comput Tomogr 19:74–80. https://doi.org/10.1016/j.jcct.2024.09.011

52.	Yamada A, Kitagawa K, Nakamura S et al (2020) Quantification of extracellular volume fraction by cardiac computed tomography for noninvasive assessment of myocardial fibrosis in hemodialysis patients. Sci Rep 10:15367. https://doi.org/10.1038/s41598-020-72417-5

53.	Mergen V, Sartoretti T, Klotz E et al (2022) Extracellular volume quantification with cardiac late enhancement scanning using dual-source photon-counting detector ct. Invest Radiol 57:406–411. https://doi.org/10.1097/RLI.0000000000000851

54.	Aquino GJ, O’Doherty J, Schoepf UJ et al (2023) Myocardial characterization with extracellular volume mapping with a first-generation photon-counting detector ct with mri reference. Radiology 307:e222030. https://doi.org/10.1148/radiol.222030

55.	Aoki S, Takaoka H, Ota J et al (2024) Strong diagnostic performance of single energy 256-row multidetector computed tomography with deep learning image reconstruction in the assessment of myocardial fibrosis. Internal Medicine 63:2499–2507. https://doi.org/10.2169/internalmedicine.2615-23

56.	Hamdy A, Kitagawa K, Goto Y et al (2019) Comparison of the different imaging time points in delayed phase cardiac ct for myocardial scar assessment and extracellular volume fraction estimation in patients with old myocardial infarction. International Journal of Cardiovascular Imaging 35:917–926. https://doi.org/10.1007/s10554-018-1513-z

57.	Gama F, Rosmini S, Bandula S et al (2022) Extracellular volume fraction by computed tomography predicts long-term prognosis among patients with cardiac amyloidosis. JACC Cardiovasc Imaging 15:2082–2094. https://doi.org/10.1016/j.jcmg.2022.08.006

58.	Emoto T, Oda S, Kidoh M et al (2021) Myocardial extracellular volume quantification using cardiac computed tomography: a comparison of the dual-energy iodine method and the standard subtraction method. Acad Radiol 28:e119–e126. https://doi.org/10.1016/j.acra.2020.03.019

59.	Egashira K, Sueta D, Tomiguchi M et al (2021) Cardiac computed tomography-derived extracellular volume fraction in late anthracycline-induced cardiotoxicity. IJC Heart and Vasculature 34:100797. https://doi.org/10.1016/j.ijcha.2021.100797

60.	Koike H, Fukui M, Treibel T et al (2024) Comprehensive myocardial assessment by computed tomography: impact on short-term outcomes after transcatheter aortic valve replacement. JACC Cardiovasc Imaging 17:396–407. https://doi.org/10.1016/j.jcmg.2023.08.008

61.	Si-Mohamed SA, Restier LM, Branchu A et al (2021) Diagnostic performance of extracellular volume quantified by dual-layer dual-energy ct for detection of acute myocarditis. J Clin Med 10:3286. https://doi.org/10.3390/jcm10153286

62.	Ishiyama M, Kurita T, Takafuji M et al (2023) The cardiac computed tomography-derived extracellular volume fraction predicts patient outcomes and left ventricular mass reductions after transcatheter aortic valve implantation for aortic stenosis. J Cardiol 81:476–484. https://doi.org/10.1016/j.jjcc.2022.12.002

63.	Deux JF, Nouri R, Tacher V et al (2021) Diagnostic value of extracellular volume quantification and myocardial perfusion analysis at ct in cardiac amyloidosis. Radiology 300:326–335. https://doi.org/10.1148/radiol.2021204192

64.	Lee HJ, Im DJ, Youn JC et al (2016) Myocardial extracellular volume fraction with dual-energy equilibrium contrast-enhanced cardiac ct in nonischemic cardiomyopathy: a prospective comparison with cardiac mr imaging. Radiology 280:49–57. https://doi.org/10.1148/radiol.2016151289

65.	Si-Mohamed SA, Congi A, Ziegler A et al (2021) Early prediction of cardiac complications in acute myocarditis by means of extracellular volume quantification with the use of dual-energy computed tomography. JACC Cardiovasc Imaging 14:2041–2042. https://doi.org/10.1016/j.jcmg.2021.04.008

66.	Ohta Y, Kitao S, Yunaga H et al (2019) Quantitative evaluation of non-ischemic dilated cardiomyopathy by late iodine enhancement using rapid kv switching dual-energy computed tomography: a feasibility study. J Cardiovasc Comput Tomogr 13:148–156. https://doi.org/10.1016/j.jcct.2018.10.028

67.	Vignale D, Palmisano A, Gnasso C et al (2023) Extracellular volume fraction (ecv) derived from pre-operative computed tomography predicts prognosis in patients undergoing transcatheter aortic valve implantation (tavi). Eur Heart J Cardiovasc Imaging 24:887–896. https://doi.org/10.1093/ehjci/jead040

68.	Ogata F, Hanatani S, Nakashima N et al (2024) Human epididymis protein 4 is a useful predictor of post-operative prognosis in patients with severe aortic stenosis. ESC Heart Fail 11:2924–2932. https://doi.org/10.1002/ehf2.14845

69.	Tavoosi A, Brito JB de O, El Mais H et al (2023) Dual versus single energy cardiac ct to measure extra cellular volume in cardiac amyloidosis: correlations with cardiac mri. IJC Heart and Vasculature 44:101166. https://doi.org/10.1016/j.ijcha.2022.101166

70.	Kidoh M, Oda S, Takashio S et al (2023) CT extracellular volume fraction versus myocardium-to-lumen signal ratio for cardiac amyloidosis. Radiology 306:e220542. https://doi.org/10.1148/radiol.220542

71.	Wang T, Ouyang B, Tang L, Li L, Liu Y (2024) Myocardial extracellular volume derived from contrast-enhanced chest computed tomography in longitudinal evaluation of cardiac toxicity in patients treated with immune checkpoint inhibitors. Quant Imaging Med Surg 14:3107–3120. https://doi.org/10.21037/qims-23-850

72.	Mirelis JG, Sánchez-González J, Zorio E et al (2017) Myocardial extracellular volume is not associated with malignant ventricular arrhythmias in high-risk hypertrophic cardiomyopathy. Revista Española de Cardiología (English Edition) 70:933–940. https://doi.org/10.1016/j.rec.2017.01.026

73.	Hammer Y, Barkan YT, Abelow A et al (2021) Myocardial extracellular volume quantification by computed tomography predicts outcomes in patients with severe aortic stenosis. PLoS One 16:e0248306. https://doi.org/10.1371/journal.pone.0248306

74.	Patel KP, Scully PR, Saberwal B et al (2024) Regional distribution of extracellular volume quantified by cardiac ct in aortic stenosis: insights into disease mechanisms and impact on outcomes. Circ Cardiovasc Imaging 17:e015996. https://doi.org/10.1161/CIRCIMAGING.123.015996

75.	Esposito A, Palmisano A, Antunes S et al (2018) Assessment of remote myocardium heterogeneity in patients with ventricular tachycardia using texture analysis of late iodine enhancement (lie) cardiac computed tomography (cct) images. Mol Imaging Biol 20:816–825. https://doi.org/10.1007/s11307-018-1175-1

76.	Morioka T, Kato S, Onoma A et al (2024) Improvement of quantification of myocardial synthetic ecv with second-generation deep learning reconstruction. J Cardiovasc Dev Dis 11:304. https://doi.org/10.3390/jcdd11100304

77.	Jiang Y, Ye J, Yang Y et al (2024) Prognostic value of measurement of myocardial extracellular volume using dual-energy ct in heart failure with preserved ejection fraction. Sci Rep 14:7504. https://doi.org/10.1038/s41598-024-58271-9

78.	Tamarappoo B, Han D, Tyler J et al (2020) Prognostic value of computed tomography–derived extracellular volume in tavr patients with low-flow low-gradient aortic stenosis. JACC Cardiovasc Imaging 13:2591–2601. https://doi.org/10.1016/j.jcmg.2020.07.045

79.	Oda S, Emoto T, Nakaura T et al (2019) Myocardial late iodine enhancement and extracellular volume quantification with dual-layer spectral detector dual-energy cardiac ct. Radiol Cardiothorac Imaging 1:e180003. https://doi.org/10.1148/ryct.2019180003

80.	Tu C, Shen H, Liu R et al (2022) Myocardial extracellular volume derived from contrast-enhanced chest computed tomography for longitudinal evaluation of cardiotoxicity in patients with breast cancer treated with anthracyclines. Insights Imaging 13:85. https://doi.org/10.1186/s13244-022-01224-5

81.	Han D, Tamarappoo B, Klein E et al (2021) Computed tomography angiography-derived extracellular volume fraction predicts early recovery of left ventricular systolic function after transcatheter aortic valve replacement. Eur Heart J Cardiovasc Imaging 22:179–185. https://doi.org/10.1093/ehjci/jeaa310

82.	Scully PR, Morris E, Patel KP et al (2020) DPD quantification in cardiac amyloidosis: a novel imaging biomarker. JACC Cardiovasc Imaging 13:1353–1363. https://doi.org/10.1016/j.jcmg.2020.03.020

83.	Suzuki M, Toba T, Izawa Y et al (2021) Prognostic impact of myocardial extracellular volume fraction assessment using dual-energy computed tomography in patients treated with aortic valve replacement for severe aortic stenosis. J Am Heart Assoc 10:e020655. https://doi.org/10.1161/JAHA.120.020655

84.	Qi RX, Shao J, Jiang JS et al (2021) Myocardial extracellular volume fraction quantitation using cardiac dual-energy ct with late iodine enhancement in patients with heart failure without coronary artery disease: a single-center prospective study. Eur J Radiol 140:109743. https://doi.org/10.1016/j.ejrad.2021.109743

85.	Monti CB, Zanardo M, Capra D et al (2023) Myocardial extracellular volume assessment at ct in hospitalized covid-19 patients with regards to pulmonary embolism. Eur J Radiol 163:110809. https://doi.org/10.1016/j.ejrad.2023.110809

86.	Ohta Y, Kishimoto J, Kitao S et al (2020) Investigation of myocardial extracellular volume fraction in heart failure patients using iodine map with rapid-kv switching dual-energy ct: segmental comparison with mri t1 mapping. J Cardiovasc Comput Tomogr 14:349–355. https://doi.org/10.1016/j.jcct.2019.12.032

87.	Wang R, Liu X, Schoepf UJ et al (2018) Extracellular volume quantitation using dual-energy ct in patients with heart failure: comparison with 3t cardiac mr. Int J Cardiol 268:236–240. https://doi.org/10.1016/j.ijcard.2018.05.027

88.	Kim NY, Im DJ, Youn JC et al (2022) Synthetic extracellular volume fraction derived using virtual unenhanced attenuation of blood on contrast-enhanced cardiac dual-energy ct in nonischemic cardiomyopathy. American Journal of Roentgenology 218:454–461. https://doi.org/10.2214/AJR.21.26654

89.	Esposito F, Mezzanotte V, Tesei C et al (2024) CT images in follicular lymphoma: changes after treatment are predictive of cardiac toxicity in patients treated with anthracycline-based or r-b regimens. Cancers (Basel) 16:563. https://doi.org/10.3390/cancers16030563

90.	Treibel TA, Bandula S, Fontana M et al (2015) Extracellular volume quantification by dynamic equilibrium cardiac computed tomography in cardiac amyloidosis. J Cardiovasc Comput Tomogr 9:585–592. https://doi.org/10.1016/j.jcct.2015.07.001

91.	Mergen V, Ehrbar N, Moser LJ et al (2024) Synthetic hematocrit from virtual non-contrast images for myocardial extracellular volume evaluation with photon-counting detector ct. Eur Radiol 34:7845–7855. https://doi.org/10.1007/s00330-024-10865-7

92.	Nowak J, Sikora M, Drabik M et al (2024) Computed tomography-derived myocardial extracellular volume fraction — a redundant by-product or a novel promising marker? Folia Med Cracov 64:129–135. https://doi.org/10.24425/fmc.2024.152173

93.	Nakato K, Oda S, Yamaguchi S et al (2025) Cardiac ct-derived quantification of myocardial extracellular volume using deep learning-based reconstruction: a feasibility study. J Cardiovasc Comput Tomogr 19:83–84. https://doi.org/10.1016/j.jcct.2024.07.008

94.	Egashira K, Sueta D, Kidoh M et al (2022) Cardiac computed tomography-derived myocardial tissue characterization after anthracycline treatment. ESC Heart Fail 9:1792–1800. https://doi.org/10.1002/ehf2.13867

95.	Kumar V, Harfi TT, He X et al (2019) Estimation of myocardial fibrosis in humans with dual energy ct. J Cardiovasc Comput Tomogr 13:315–318. https://doi.org/10.1016/j.jcct.2018.12.004

96.	Takahashi M, Takaoka H, Yashima S et al (2024) Extracellular volume fraction by computed tomography predicts prognosis after transcatheter aortic valve replacement. Circulation Journal 88:492–500. https://doi.org/10.1253/circj.CJ-23-0288

97.	Nishigake D, Yamasaki Y, Hida T et al (2024) Influence of cardiac cycle on myocardial extracellular volume fraction measurements with dual-layer computed tomography. Quant Imaging Med Surg 14:4714–4722. https://doi.org/10.21037/qims-23-1647

98.	Yamasaki H, Kondo H, Shiroo T et al (2024) Efficacy of computed tomography-based evaluation of myocardial extracellular volume combined with red flags for early screening of concealed cardiac amyloidosis in patients with atrial fibrillation. Circulation Journal 88:1167–1175. https://doi.org/10.1253/circj.CJ-23-0948

99.	Liang J, Li H, Xie J et al (2022) Iodine-based extracellular volume for evaluating myocardial status in patients undergoing percutaneous coronary intervention for acute myocardial infarction by using dual-layer spectral detector computed tomography: a comparison study with magnetic resonance. Quant Imaging Med Surg 12:4502–4511. https://doi.org/10.21037/qims-21-1103

100.	Oda S, Kidoh M, Takashio S et al (2020) Quantification of myocardial extracellular volume with planning computed tomography for transcatheter aortic valve replacement to identify occult cardiac amyloidosis in patients with severe aortic stenosis. Circ Cardiovasc Imaging 13:e010358. https://doi.org/10.1161/CIRCIMAGING.119.010358

101.	Abadia AF, van Assen M, Martin SS et al (2020) Myocardial extracellular volume fraction to differentiate healthy from cardiomyopathic myocardium using dual-source dual-energy ct. J Cardiovasc Comput Tomogr 14:162–167. https://doi.org/10.1016/j.jcct.2019.09.008

102.	Gnasso C, Pinos D, Schoepf UJ et al (2024) Impact of reconstruction parameters on the accuracy of myocardial extracellular volume quantification on a first-generation, photon-counting detector ct. Eur Radiol Exp 8:70. https://doi.org/10.1186/s41747-024-00469-7

103.	Kidoh M, Oda S, Takashio S et al (2020) Assessment of diffuse ventricular fibrosis in atrial fibrillation using cardiac ct-derived myocardial extracellular volume fraction. JACC Clin. Electrophysiol. 6:1573–1575

104.	Sueta D, Kidoh M, Oda S et al (2021) Usefulness of cardiac computed tomography in the diagnosis of anti-cancer therapy-related cardiac dysfunction - consistency with magnetic resonance imaging. Circulation Journal 85:393–396. https://doi.org/10.1253/CIRCJ.CJ-20-1288

105.	Kurita Y, Kitagawa K, Kurobe Y et al (2016) Estimation of myocardial extracellular volume fraction with cardiac ct in subjects without clinical coronary artery disease: a feasibility study. J Cardiovasc Comput Tomogr 10:237–241. https://doi.org/10.1016/j.jcct.2016.02.001

106.	Takafuji M, Kitagawa K, Nakamura S et al (2020) Feasibility of extracellular volume fraction calculation using myocardial ct delayed enhancement with low contrast media administration. J Cardiovasc Comput Tomogr 14:524–528. https://doi.org/10.1016/j.jcct.2020.01.013

107.	Goto H, Takaoka H, Ota J et al (2024) Computed tomography evaluation of extracellular volume for predicting prognosis in patients with severe renal dysfunction on dialysis. J Clin Med 13:7561. https://doi.org/10.3390/jcm13247561

108.	Rosenfeld R, Riondino S, Cerocchi M et al (2024) Extracellular volume measured by whole body ct scans predicts chronic cardiotoxicity in breast cancer patients treated with neoadjuvant therapies based on anthracyclines: a retrospective study. Breast 76:103755. https://doi.org/10.1016/j.breast.2024.103755

109.	Oguni T, Takashio S, Kuyama N et al (2024) Clinical characteristics of patients with high extracellular volume fraction evaluated by cardiac computed tomography for coronary artery evaluation. European Heart Journal Open 4:oeae036. https://doi.org/10.1093/ehjopen/oeae036

110.	Chiocchi M, Cerocchi M, Di Tosto F et al (2023) Quantification of extracellular volume in ct in neoadjuvant chemotherapy in breast cancer: new frontiers in assessing the cardiotoxicity of anthracyclines and trastuzumab. J Pers Med 13:199. https://doi.org/10.3390/jpm13020199

111.	Dubourg B, Dacher JN, Durand E et al (2021) Single-source dual energy ct to assess myocardial extracellular volume fraction in aortic stenosis before transcatheter aortic valve implantation (tavi). Diagn Interv Imaging 102:561–570. https://doi.org/10.1016/j.diii.2021.03.003

112.	Li N, Zhang X, Gu J et al (2024) Quantitating myocardial fibrosis using extracellular extravascular volume determined from computed tomography myocardial perfusion imaging. BMC Med Imaging 24:40. https://doi.org/10.1186/s12880-024-01226-3

113.	Emoto T, Kidoh M, Oda S et al (2020) Myocardial extracellular volume quantification in cardiac ct: comparison of the effects of two different iterative reconstruction algorithms with mri as a reference standard. Eur Radiol 30:691–701. https://doi.org/10.1007/s00330-019-06418-y
